# Trophic resource partitioning drives fine‐scale coexistence in cryptic bat species

**DOI:** 10.1002/ece3.7004

**Published:** 2020-11-11

**Authors:** Roberto Novella‐Fernandez, Carlos Ibañez, Javier Juste, Elizabeth L. Clare, C. Patrick Doncaster, Orly Razgour

**Affiliations:** ^1^ School of Biological Sciences University of Southampton Southampton UK; ^2^ Estación Biológica de Doñana (CSIC) Sevilla Spain; ^3^ CIBER Epidemiology and Public Health (CIBERESP) Madrid Spain; ^4^ School of Biological and Chemical Sciences Queen Mary University of London London UK; ^5^ Biosciences University of Exeter Exeter UK

**Keywords:** bats, cryptic species, DNA metabarcoding, interspecific competition, molecular diet analysis, *Myotis nattereri* species complex, species coexistence, trophic partitioning

## Abstract

Understanding the processes that enable species coexistence has important implications for assessing how ecological systems will respond to global change. Morphology and functional similarity increase the potential for competition, and therefore, co‐occurring morphologically similar but genetically unique species are a good model system for testing coexistence mechanisms. We used DNA metabarcoding and high‐throughput sequencing to characterize for the first time the trophic ecology of two recently described cryptic bat species with parapatric ranges, *Myotis escalerai* and *Myotis crypticus*. We collected fecal samples from allopatric and sympatric regions and from syntopic and allotopic locations within the sympatric region to describe the diets both taxonomically and functionally and compare prey consumption with prey availability. The two bat species had highly similar diets characterized by high arthropod diversity, particularly Lepidoptera, Diptera and Araneae, and a high proportion of prey that is not volant at night, which points to extensive use of gleaning. Diet overlap at the prey item level was lower in syntopic populations, supporting trophic shift under fine‐scale co‐occurrence. Furthermore, the diet of *M. escalerai* had a marginally lower proportion of not nocturnally volant prey in syntopic populations, suggesting that the shift in diet may be driven by a change in foraging mode. Our findings suggest that fine‐scale coexistence mechanisms can have implications for maintaining broad‐scale diversity patterns. This study highlights the importance of including both allopatric and sympatric populations and choosing meaningful spatial scales for detecting ecological patterns. We conclude that a combination of high taxonomic resolution with a functional approach helps identify patterns of niche shift.

## Graphical abstract

We used DNA metabarcoding, high‐throughput sequencing, and a functional approach to test the role of trophic partitioning in enabling species coexistence across spatial scales through characterizing the trophic ecology of two cryptic bat species, Myotis escalerai and Myotis crypticus, in sympatric versus allopatric locations. Although the two species had similar diets characterized by high arthropod diversity, diet overlap at the prey‐item level was lower in locally sympatric than allopatric locations; and the functional analysis suggested that this trophic shift may be driven by a change in foraging mode from gleaning to aerial hawking. Our findings show that fine‐scale coexistence mechanisms can contribute to maintaining broad‐scale diversity patterns.

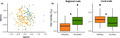

## 1 INTRODUCTION

Understanding the processes that enable species coexistence is a key theme of ecology with important implications for interpreting diversity patterns and predicting how systems respond to global change (Valladares et al., 2015). Interspecific competition is thought to have a major influence on community structure for many taxonomic groups (Weiher & Keddy, 1999). Niche theory (Chase & Leibold, 2003; Chesson, 2000; Letten et al., 2017) asserts that species coexistence is promoted through differential use of resources driven by functional differences between species, which results in communities that tend to be assembled by functionally dissimilar species (Schoener, 1974). This has been shown in numerous cases, including fish (Ross, 1986), shorebirds (Bocher et al., 2014), and rodent communities (Codron et al., 2015). Alternatively, community structure and coexistence, primarily in sessile organisms, have been often explained through neutral processes, such as dispersal or stochasticity (The neutral theory of biodiversity and biogeography; Hubbell, 2001). This framework has been often used as a null model to evaluate whether observed patterns deviate from neutral expectations (Alonso et al., 2006; McGill et al., 2006). Yet, some studies of mobile organisms have failed to identify evidence of resource partitioning (e.g., Luiselli, 2008), suggesting that in some cases biotic interactions only play a minor role in governing community assembly, perhaps because resources are not limiting (Salinas‐Ramos et al., 2020), and therefore, neutral processes likely play a more important role.

Morphologically similar species pose a challenge for understanding mechanisms of coexistence from a niche theory perspective because they are more likely to be functionally similar, and therefore less likely to be able to use resources in a different way, a prerequisite for resource partitioning (Weiher & Keddy, 1999). Consequently, considerable attention has been given to understanding resource partitioning among morphologically identical (cryptic) or similar co‐occurring species (e.g., Gabaldón et al., 2013; Jiang et al., 2008; Razgour et al., 2011). Many studies have focused on the trophic dimension, an important aspect of species’ ecological niche (Schoener, 1974). DNA metabarcoding and high‐throughput sequencing (molecular diet analysis) approaches helped overcome many of the limitations of traditional morphological methods (Sousa et al., 2019), opening the door to new opportunities for studying mechanisms of species coexistence (Arrizabalaga‐Escudero et al., 2018; Krüger et al., 2014; Razgour et al., 2011). However, the majority of coexistence studies focus on only sympatric populations, preventing an evaluation of how the presence of a competitor may change resource use, thus limiting the power of inferences (Salinas‐Ramos et al., 2020). Moreover, most studies also focus on diet only, and disregard prey selection relative to prey availability or resource limitation (Salinas‐Ramos et al., 2020). Accounting for prey selection (e.g., Rytkönen et al., 2019) can provide a more complete picture of consumer trophic preferences (Lawlor, 1980).

The processes that govern community assembly, including coexistence mechanisms, vary with spatial scale (Lewis et al., 2015; Snyder & Chesson, 2004; Viana & Chase, 2019), yet spatial scale is rarely considered in coexistence studies (Hart et al., 2017). A better understanding of the scale of coexistence mechanisms and how different processes interact is important for both basic and applied ecology (Peixoto et al., 2018).

**Figure 1:**
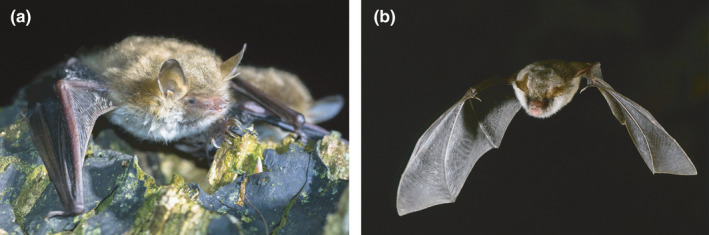
*Myotis crypticus*(a) and*Myotis escalerai*(b) in Spain. Photographs taken by Roberto Novella‐Fernandez and Daniel Fernandez‐Alonso

This study aims to identify whether trophic ecology enables morphologically similar species to coexist across spatial scales. We focus on two recently described insectivorous bat species whose trophic ecology has not been studied to date, *Myotis crypticus* and *Myotis escalerai* (Figure 1). Both species forage in forests and are morphologically nearly identical, only distinguishable by a small difference in the uropatagium and its terminal row of hairs (Juste et al., 2019). These bats are restricted to the Western Mediterranean Basin, where they overlap across the north of the Iberian Peninsula, but at the fine‐scale are known to co‐occur only in a few locations (Juste et al., 2019). Phylogeographic analysis and species distribution modeling suggest that their ranges have been shaped by competition (Razgour et al., 2015). These bats therefore provide an excellent case study for understanding mechanisms of coexistence among morphologically similar species. We use DNA metabarcoding and high‐throughput sequencing to characterize the trophic ecology of *M. crypticus* and *M. escalerai* by analyzing their taxonomic and functional diets and their prey selection relative to prey availability in sympatry versus allopatry at both fine and regional spatial scales. Given their near identical morphology and echolocation calls, the overall trophic niches of the two bats are expected to be similar and niche overlap should be high. We hypothesize that if resource partitioning is the main process facilitating coexistence: (a) trophic niche overlap and diet similarity will be higher in allopatry than sympatry (e.g., Klawinski et al., 1994), and (b) differences in trophic niche overlap will be most pronounced at the fine spatial scale where individuals of the two species directly co‐occur.

## 2 METHODS

### 2.1 Sampling design

**Figure 2:**
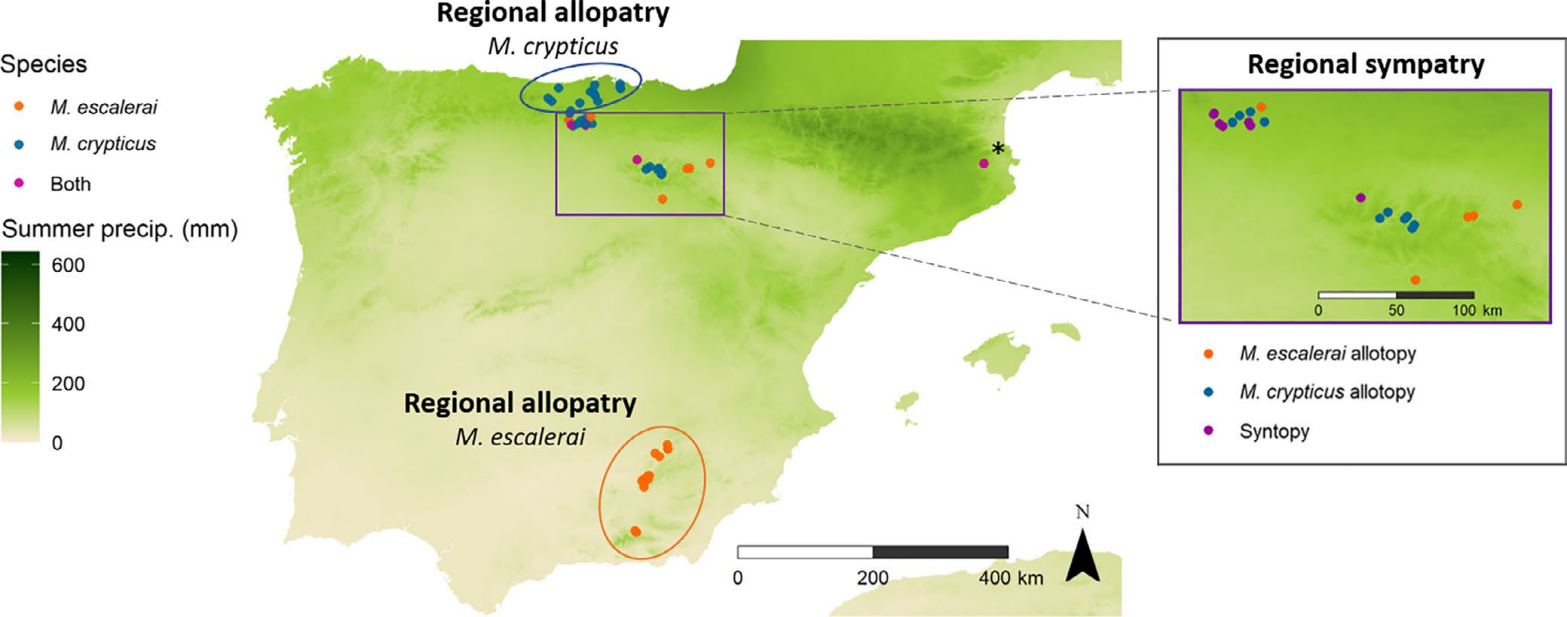
Sampling locations overlaying summer precipitation across Spain, with gradient from dry Mediterranean to wet Atlantic. Ovals encompass the two allopatric regions (Granada and Jaen for*M. escalerai*and northern Cantabria for*M. crypticus*). The rectangle encompasses the sympatric region (La Rioja and the Mediterranean climatic zone at the south of Cantabria). Insert shows the sympatric region with syntopic (red) versus allotopic (blue and orange) locations. Black star near the sympatric location at the eastern side of the main map in Catalonia denotes a swarming site and was excluded from the analysis

Sampling took place in the Iberian Peninsula, focusing on two sympatric regions in the north where both *Myotis escalerai* and *Myotis crypticus* are found (La Rioja‐Soria and southern Cantabria), and two allopatric regions: the south (Andalusia: Jaen and Granada), where only *M. escalerai* is found, and the north Atlantic coast (northern Cantabria), where only *M. crypticus* is found. Additionally, we sampled a single swarming site in Catalonia, where the two species use during the autumn mating season (Figure 2). Within each region, 9–24 locations were sampled based on suitable habitat and accessibility, using monofilament mist nets and a harp trap placed over water sources, forest paths and cave entrances. The sampling period extended from June to September 2017, for a total of 68 sampling nights (Table S1 for list of sampling locations). Captured bats were kept in individual cotton bags for up to 1 hr. We collected fecal samples from the cotton bags for diet analysis, and biopsy punches (3 mm) from the wing membrane of the bats to confirm species identification. Dropping samples and wing biopsies were stored for each bat individually in absolute and 70% ethanol, respectively. Bat sampling was carried out under local permits and ethical approval from the University of Southampton (study ID: 26627).

We sampled the arthropod community in bat sampling locations using vegetation sweeping (Barclay, 1991; Swift & Racey, 2002) to assess bat prey selectivity relative to prey availability (Jones, 1990; Kunz, 2009). We chose vegetation sweeping because of the expected low flight and gleaning behavior of the species based on their morphology and echolocation calls, and the foraging behavior of the morphologically similar better studied member of the cryptic species complex, *Myotis nattereri* (de Jong, 1995). During bat sampling nights, we set linear transects in each habitat type in the sampling location and swept the vegetation along each transect. After 10 sweeps, we collected the capture into a plastic bag and moved five steps further without sampling to increase spatial representativeness. Each sampling unit of 10 sweeps and five steps forward was repeated 5–10 times until the capture size was considered representative (>100 individuals). Transect length ranged between 30 and 80 m. Arthropod specimens captured were separated from vegetation remains in the field and stored in 70% ethanol. A total of 43 sweeping samples from 23 locations with at least three bat individuals were selected (Table S1).

### 2.2 DNA extractions and species confirmation

Bat species identity was confirmed in the Estación Biológica de Doñana Laboratory of Molecular Ecology (LEM, EBD‐CSIC, Spain). DNA was extracted from wing biopsy punches through precipitation with isopropanol. Part of the hypervariable region of the mtDNA control region was amplified using the primers CSBC‐F 5′‐CCTCTTAAATAAGACATCTCGATGG‐3′ (Wilkinson & Chapman, 1992) and HV2‐Mna‐R 5′‐ATGCGTGCGTGTGTAATGTC‐3′ (Garcia‐Mudarra et al., In Press). Species specific differential amplification patterns for this primer set were used to confirm species identity through gel electrophoresis (Garcia‐Mudarra et al., In Press).

DNA was extracted from all bat dropping samples using the Qiagen DNA stool mini kit, following the protocol in Zeale et al. (2011). From the 43 selected sweeping samples, all arthropod individuals (*N* = 8,366) were first identified morphologically to taxonomic order. Subsequently, whole specimens, if smaller than a drosophila, or a specimen part (leg or head) if larger, were separated out, dried and pooled together for DNA extraction. Arthropod DNA was extracted using the NucleoSpin DNA Insect kit with up to 35 mg of sample dry weight in each tube. Larger samples were split into several tubes. The following modifications were applied to the kit extraction protocol: In steep 2, vortex for 20 min in the MN Bead Tube Holder on a Vortex‐Genie at maximum speed; after steep 3, pipette 550 µl of clean supernatant in to a new 2 ml Eppendorf, centrifuge again at 20,000 *g* for 2 min and continue with steep 4; in steep 6, centrifuge for 3 min; in steep 7, add 50 µl of ddH_2_0 and incubate for 3 min.

### 2.3 High‐throughput sequencing

Both dropping and sweeping samples were sequenced in the Bart's and the London Genome Centre, London, UK. DNA extracts were checked for quality and concentration on a TapeStation D1000. Two sets of primers were used together in order to reduce primer taxonomic bias (Alberdi et al., 2018), especially given the high diversity of prey types expected in the diet, ZBJ (Zeale et al., 2011) Forward: ZBJ‐ArtF1c 5′‐AGATATTGGAACWTTATATTTTATTTTTGG‐3′ and Reverse: ZBJ‐ArtR2c 5′‐WACTAATCAATTWCCAAATCCTCC‐3′, and ANML (Jusino et al., 2019) Forward: LCO1490 5′‐GGTCAACAAATCATAAAGATATTGG‐3′ and Reverse: CO1‐CFMRa 5′‐GGWACTAATCAATTTCCAAATCC‐3′. For the ZBJ amplicon each 15 μl PCR reaction used 7.5 μl of Multiplex PCR mastermix (QIAGEN, Germany), 0.25 μL of each primer (10 μM), 5 μL H2O and 2 μl template DNA. Negative and positive controls were included in PCR reactions and later sequencing. The thermal cycling protocol was as follows: 95°C for 15 min, 34 cycles of 94°C for 40s, 40°C for 1 min, 72°C for 30s, followed by a final extension of 72°C for 5 min. ANML regions were amplified in 15 µl reactions following published protocols (Jusino et al., 2019). All products were visualized on a 1.5% agarose gel. Products were tagged using Fluidigm barcodes and checked on a TapeStation D1000 before pooling and sequencing on an Illumina MiSeq using paired end (2 × 250 bp) chemistry (Illumina, San Francisco, USA). We used two technical PCR replicates to reduce biases associated with PCR stochasticity. This led to each sample being sequenced four times (combination of two primer and two PCR replicates).

### 2.4 Bioinformatics

Sequencing runs were merged using USEARCH (Edgar, 2010) and primers and adaptors removed using cutadapt (Martin, 2011). Sequences were processed on the mBRAVE platform (http://www.boldsystems.org/bin) (Ratnasingham & Hebert, 2007) setting the following parameters: Min QV = 0 qv, Min Length = 100 bp, Max Bases with Low QV (<20) = 75%, Max Bases with Ultra Low QV (<10) = 75%, ID Distance Threshold = 1.5%, Exclude from OTU Threshold = 3%, Minimum OTU Size = 1, OTU Threshold = 2%. Sequences were compared with the BOLD reference libraries SYS‐CRLINSECTA and SYS‐CRLNONINSECTARTH to established Barcode Index Numbers (BINs). BINs are a type of Operational Taxonomic Unit (OTU) integrated in the BOLD system with advantages over traditional OTUs, such as being unique and stable (Ratnasingham & Hebert, 2013). We used BIN identity as a proxy of taxonomic prey item unit.

After obtaining BIN (prey item) composition per sample and run, we removed singletons, that is, BINs that only had a single read per run and sample, because they are likely to be PCR or sequencing errors (Alberdi et al., 2018). We set the threshold for the minimum number of reads needed to be present in each sample to retain a BIN identification to two (singletons removed) because this threshold resulted in highest similarity in arthropod composition in sweeping samples between molecular and morphological identification methods (range of thresholds tested: 1–5 minimum reads/sample). We controlled for potential contamination during the extraction and sequencing process by removing the BINs present in extraction/sequencing blanks from samples in the same extraction/sequencing run that had a similar number of these reads to the blanks (less than 10 times more reads in samples than in blank). To assess impact of BIN removal, all analyses were run without removing the BINs potentially resulting from contamination. We found that BIN removal did not affect the study results.

We used two alternative approaches to combine data from PCR replicates. In the first, the additive criteria, BIN composition from both PCR replicates of each sample were added together. In the second, the conservative approach, only BINs that appeared in both PCR replicates were considered (Alberdi et al., 2018). Under this second criteria only samples in which the four runs contained a minimum number of reads (>100) were considered given that a failed sequencing run in a sample would lead to a null composition for both of the PCR replicates of a primer (52 samples, Table S3). Finally, we combined taxa recovered from both primers to obtain the prey composition per dropping sample for downstream ecological analysis. Duplicated BINs in the same sample coming from different PCR replicates or primers were removed. A flow chart describing the methods is shown in Fig. S1. Ecological results from both approaches were very similar, thus we present results based on the additive approach (See Fig. S9 for diet based on the conservative approach).

### 2.5 Characterizing the diets of the two bat species

The contribution of different elements to the diet for a set of samples was quantified using weighted Percent of Occurrence (wPOO), which measures the relative occurrence of diet elements (prey items/OTUs/BINs) in a set of samples considering first their relative proportion per sample (Deagle et al., 2019). For example, a prey item found in a sample with 9 other prey items will be interpreted to contribute in the diet 1/10 of what it would if it was the only prey item present. Contributions to diet based on the two other commonly used metrics: Percent of Occurrence (POO) and Relative Read Abundance (RRA) (Deagle et al., 2019), are shown in (Fig. S2). We tested for differences in the number of BINs per sample between bat species for each of the orders that constitute at least 10% of the diet of either one of the bat species (Araneae, Diptera, Lepidoptera, and Hemiptera), using negative binomial generalized linear models (GLMs; in R) to fit data structure based on the distribution of model residuals. We measured order level and prey species level (BIN‐level) diet composition overlap between bat species using Pianka's measure of niche overlap (O_jk_) (R package: EcoSimR (Gotelli et al., 2015). We tested with an ANOSIM test (R package vegan (Oksanen et al., 2019)) whether Jaccard distance in BIN composition was greater between than within bat species. The ANOSIM statistic R is based on the difference of mean ranks between and within groups, with a range between −1 and +1. A value of zero indicates that the group does not explain compositional differences. We visualized ordination of samples depending on their BIN composition with Non‐Metric Multidimensional Scaling (NMDS, R package vegan: Oksanen et al., 2019). We calculated Levins’ (1968) standardized measure of niche breadth (B_A_) at the prey species (BIN) level for each bat species.

### 2.6 Functional diet assessment

Prey items were classified based on the literature (outlined in Table S2) and an expert entomological taxonomist into three functional categories: non‐volant, not actively volant, nocturnally volant. Categorization depended on their mobility and type of activity, reflecting their likelihood of being captured by gleaning or aerial hawking (Data file S1, Table S2). The categorization was done at family or finer taxonomic level by checking the literature for data on daily activity patterns of each family and presence in nocturnal light traps (Table S2 for criteria used). The non‐volant category included wingless arthropod groups (Araneae, Isopoda and wingless insects such as some members of Blattodea, Orthoptera). The not actively volant category included those able to fly but unlikely to have been captured by the bat through aerial hawking because they are not active fliers, either at night (diurnal Diptera), or not active fliers in general (e.g., Hemiptera, some Blattodea, Orthoptera and Coleoptera). The nocturnally volant category comprised arthropods with aerial and nocturnal activity and therefore likely to be captured by aerial hawking (e.g., non‐Ropalocera Lepidoptera, nocturnal Diptera, Neuroptera, Ephemeroptera, Trichoptera). This classification represents the likelihood of being captured by gleaning or aerial hawking rather than direct inference of the capture mode because nocturnally active aerial prey can also be captured by gleaning when resting on vegetation and not active nocturnal fliers could also be captured in the air (e.g., ballooning in spiders). Once all prey items were classified into functional groups, we obtained the functional diet of both bat species using weighted percent of occurrence (wPOO), and compared the percentage of not nocturnally volant (including both non‐volant and not actively volant categories) per sample between bat species using a linear model.

### 2.7 Trophic niche overlap in allopatry versus sympatry across spatial scales

Locations from Andalusia (Mediterranean climate) and northern Cantabria (Atlantic climate) were classified as regionally allopatric. Locations from La Rioja and southern Cantabria (climatically Mediterranean to sub‐Atlantic) as regionally sympatric (based on data from Razgour et al., (2019) and EBD records). At the fine‐scale within the sympatric regions, we classified locations as syntopic (both species co‐occurring) or allotopic (species not co‐occurring) depending on whether they were within 3 km of records of the other species. Distances were set based on a conservative estimation of the home‐range distance of the better studied cryptic congener *M. nattereri* (Boye & Dietz, 2005). The swarming location in Catalonia was removed from the fine‐scale analysis because bats gather in swarming sites from distances of up to 60 km from their colonies for the purpose of breeding rather than foraging (Rivers et al., 2005), and therefore, it is unclear whether those individuals forage in sympatry (Table S1 for sampling locations and their broad and fine‐scale sympatry category).

To identify differential use of certain prey orders and functional groups, we tested separately for allopatry and sympatry whether (a) the number of BINs per sample for each of the main arthropod orders, and (b) the percentage of not nocturnally volant functional groups differed between bat species. We used negative binomial zero inflated GLMs and a linear model respectively. We run separate models for broad and fine spatial scales given that both syntopy and allotopy treatments are within regional sympatry. Cases where resource (prey order or functional group) use was different between bat species when sympatric but not when allopatric were regarded as evidence of resource partitioning. We measured prey species (BIN) level niche overlap (O_jk_) between bat species in sympatry and in allopatry, and tested, using null models (R package ecosimR) whether overlap was lower or higher than random in sympatry versus allopatry. We tested whether O_jk_ differed between sympatric and allopatric regions and syntopic and allotopic locations by pooling the diet composition of each bat species per site and measuring O_jk_ between pairs of sites. At the regional scale we used a Gaussian Hurdle model due to the high density of zeroes in overlap values. At the local scale we used a linear model with log transformed values of O_jk_ to meet assumptions of normal distribution. All statistical analysis was carried out in R (R core team, 2020).

### 2.8 Prey consumption relative to availability

To assess the representativeness of arthropod availability sampling, we checked for each dropping sample the proportion of BINs that appear in the sweeping samples from the same site. We retained only the sites where prey availability sampling was considered most representative because at least 20% of the diet prey items in the site were found in sweeping samples. For each of these sites, we quantified the relative availability of each arthropod order and functional group using weighted percent of occurrence (wPOO) after pooling together sweeping samples from the different habitats. Similarly, we obtained bat diet composition (wPOO) per site for each arthropod order and functional group by pooling diet composition of all individual bats. Then, we subtracted from the bat diet wPOO the prey availability wPOO to obtain prey arthropod and functional group selection per site. A higher proportion of a given arthropod order in the diet than in sweeping samples indicates the bats may be preferentially consuming this resource, based on prey availability at the sampled strata. We calculated whether confidence intervals of selection values across sites are above zero.

### 2.9 Testing primer performance and representation of the DNA metabarcoding approach

For each arthropod order we described the number and proportion of BINs identified by each primer. Morphological identification of the arthropod communities allowed us to compare the performance of the primers and metabarcoding approaches. We compared the presence of orders in each sweeping sample based on molecular and morphological identification to determine whether metabarcoding offers a good estimation of arthropod community composition.

## 3 RESULTS

We analyzed a total of 138 dropping samples for *Myotis escalerai* and 90 for *Myotis crypticus* from 49 locations, 26 of which were in the broad‐scale allopatric regions and 23 in sympatric regions. Within the sympatric regions (La Rioja and southern Cantabria), 91 samples were classified as allotopic and 28 as syntopic (Figure 2, Table 1; Table S1). Sample sizes were limited by the small number of known syntopic locations for these species. We recovered a total of 2,859,300 reads (Table S3 for details) from the 228 dropping samples for the four combinations of PCR replicates and primers (1,403,636 from ANML1 and 1,455,664 from ZBJ). These reads were associated into 1,461 different BINs, 941 for *M. escalerai* and 851 for *M. crypticus*. Based on BINs present in extraction blanks, we removed a total of 8 BINs from 10 dropping samples (Table S4). Based on the BINs present in sequencing blanks, we removed for the ANML primers 6 BINs from 66 dropping samples, and for the ZBJ primers, 24 BINs from 95 samples (Table S5).

**Table 1:** Number of bat dropping samples, sweeping samples, and locations for each bat species by allopatry/sympatry classification at broad (regional) and fine (local) spatial scales

		Total	Broad‐scale allopatric	Broad‐scale sympatric	Allotopic	Syntopic
Dropping samples	*M. escalerai*	138	82	56	46	6
	*M. crypticus*	90	18	72	45	22
Sweeping samples	*M. escalerai*	13	5	8	3	5
	*M. crypticus*	15	2	13	6	7
Locations		49	26	23	14	8

### 3.1 Characterizing the diet of *M. escalerai* and *M. crypticus*

**Figure 3:**
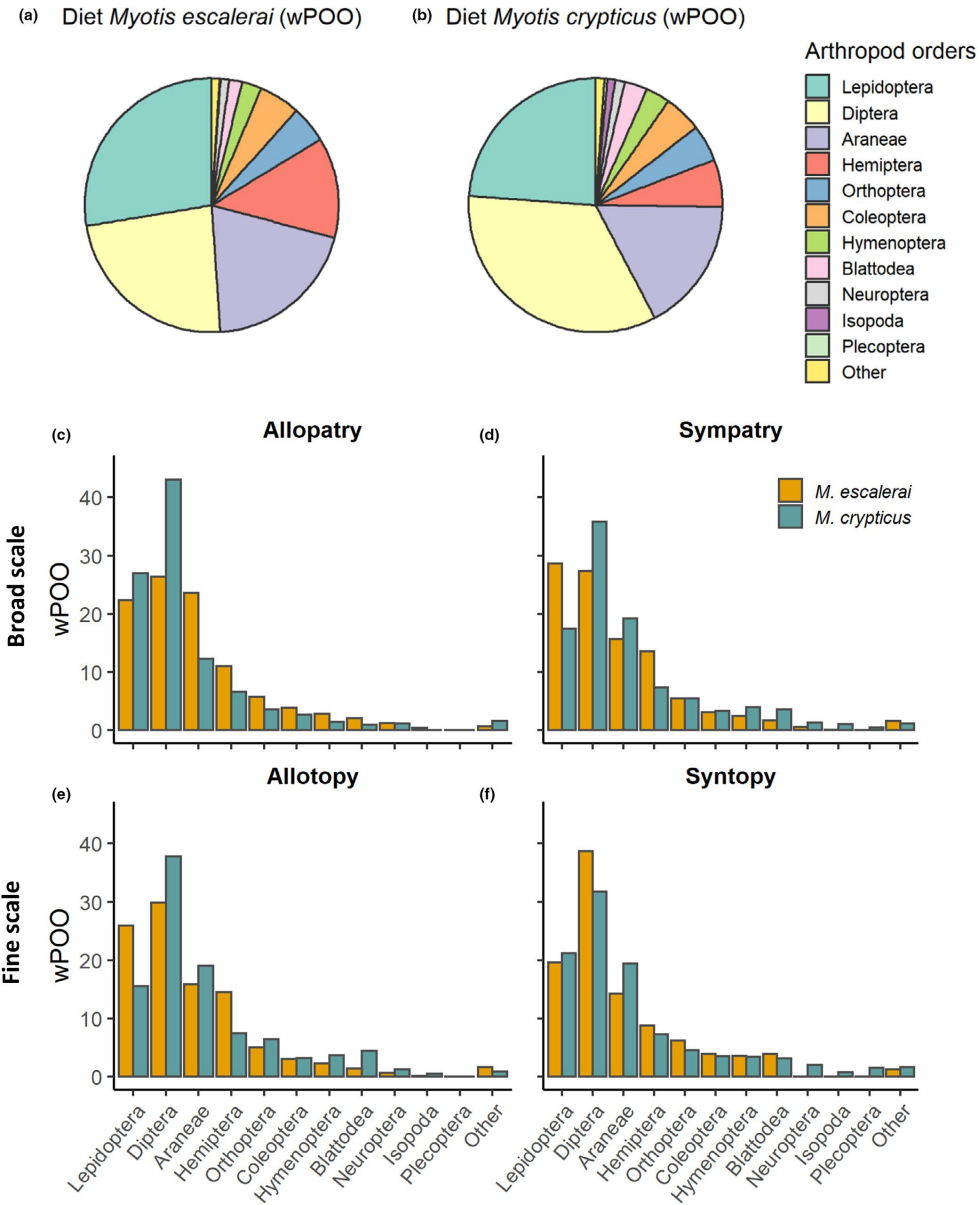
Overall diet composition of*M. escalerai*and*M. crypticus*using*w*eighted Percent of Occurrence (wPOO) (a, b). Dietary composition by scale of allopatry/sympatry (broad‐scale: c, d, fine‐scale: e, f)

A total 19 arthropod orders were obtained based on the 1,461 BINs (Supplementary Data file S1 for list of prey items obtained for each bat species). The diets of *M. escalerai* and *M. crypticus* were characterized by high arthropod diversity, and were composed mostly of the orders Lepidoptera (*M. escalerai* = 26.6% wPOO; *M. crypticus* = 23.7%), Diptera (24.8%; 33.2%), Araneae (20.7%; 17.2%), but also included Hemiptera (11.8%; 6.2%), Coleoptera (4.8%; 5.1%), and Orthoptera (4.3%; 4.8%), among others (<5%) (Figure 3a‐b; Fig. S2 for diet composition based on POO and RRA measures). The most common prey species consumed by *M. escalerai* were *Philodromus dispar*, *Xysticus ferrugineus/audax*, *Chorthippus vagans*, *Metasia* sp., and *Ectobius pallidus,* while the most common prey species consumed by *M. crypticus* were *Philodromus dispar,* *Ectobius pallidus*, *Araneus diadematus*, *Delia platura*, and *Chorthippus vagans*. Hence, the two bats shared three of the top five common prey species. Diet composition at the prey order level was very similar between bat species (O_JK_ = 0.98, above 1,000 null models). However, there were differences in the number of BINs per sample of Diptera, which was lower in *M. escalerai* (5.27 versus 6.75) (Negative binomial GLM: *z*_1,226_ = −2.03, *p* = .042), and Hemiptera, which was higher in *M. escalerai* (2.09 versus 1.68) (Negative binomial GLM: *z*_1,226_ = 2.85, *p* = .004 Fig. S3).

**Figure 4:**
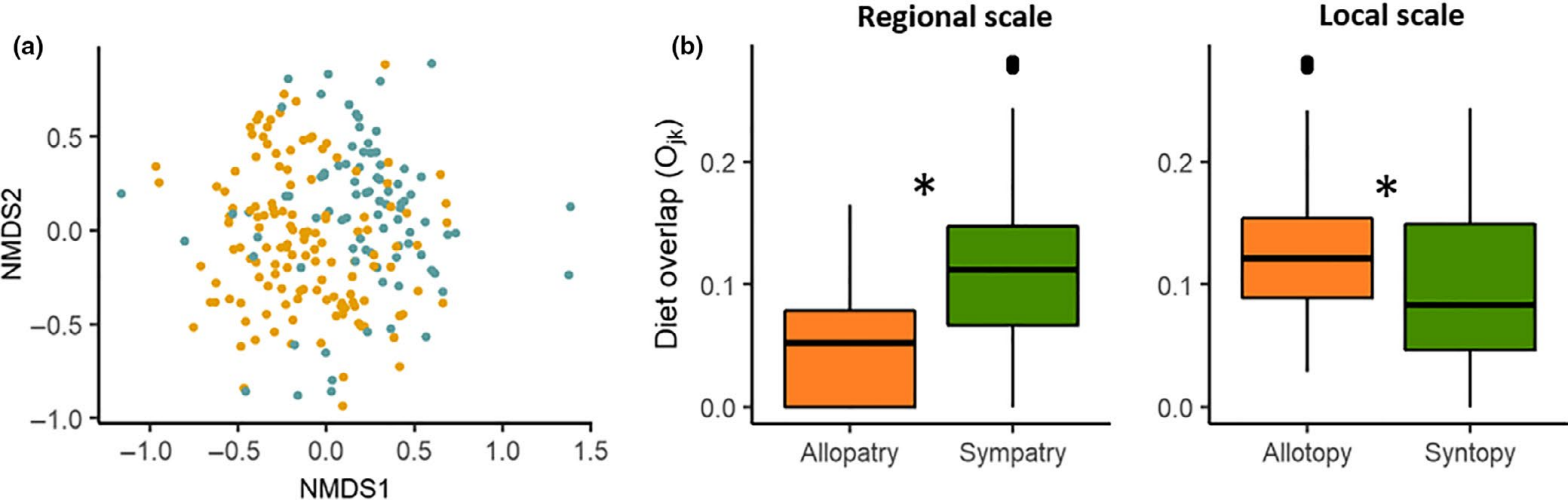
(a) Non‐Metric Multidimensional Scaling ordination of individual bat samples based on their BIN composition, with*M. escalerai*samples in yellow and*M. crypticus*in blue. (b) Pianka's measure of niche overlap (O_JK_) between the two bat species in allopatric versus sympatric locations at the regional (left) and local (right) scales. Replicates are values of overlap between pairs of locations of different bat species. Star denotes significant differences between groups

At the prey species (BIN) level, Levins’ niche breadth was similar for both species, B_A_ = 0.17 for *M. escalerai* and B_A_ = 0.19 for *M. crypticus*. Niche overlap between species was higher than expected by chance (O_JK_ = 0.71, above 95% of 1,000 null models). The samples from the two bat species showed some differences in prey item composition in NMDS ordination space (Figure 4a, Stress: 0.25, *k* = 3, non‐metric fit *R* ^2^ = 0.934, Linear fit, *R* ^2^ = 0.532). An analysis of similarity confirms that distance in prey item composition among samples is greater between species than within species (ANOSIM R statistic: 0.10, *p* = .001).

### 3.2 Trophic partitioning in sympatric versus allopatric locations

At the arthropod order level, there is no clear pattern of shift from high similarity in order composition between species to differential use in sympatry at any of both spatial scales (O_JK_ regional allopatry = 0.88, O_JK_ regional sympatry = 0.96, >1,000 null models, Figure 2c‐d; O_JK_ allotopy = 0.95, O_JK_ syntopy = 0.98, >1,000 null models, Figure 3e‐f). When examining the number of BINs of the main arthropod orders per sample, there were differences between bat species between the allopatric regions for Araneae and Hemiptera, which were both higher in *M. escalerai* (*M. escalerai* = 4.00, 1.65, *M. crypticus* = 2.16, 0.55 respectively), and for Lepidoptera, which was higher in *M. crypticus* (4.6, 11.94) (Negative binomial GLM: *df* = 1,98, *p* < .05). In the sympatric region, the higher number of Hemiptera in *M. escalerai* holds (*M. escalerai* = 2.50, *M. crypticus* 1.50), and in Lepidoptera there is a shift whereby is *M. escalerai* the one that consumes a higher number (*M. escalerai* = 6.78, *M. cryptius* = 4.09, Negative binomial GLM: *p* < .05). At the fine‐scale, within the sympatric region, the only difference found between the bat species was the higher number of BINs per sample of Hemiptera (2.67, 1.49) (Negative binomial GLM: z_1,89_ = −2.68, *p* = .007) and Lepidoptera (6.70, 3.64) in *M. escalerai* in allotopic locations (Negative binomial GLM: z_1,89_= −2.92, *p* = .004). There were no differences in arthropod orders consumed between the bat species in syntopic locations (Negative binomial GLM: *p* > .05; Fig. S4).

At the prey species (BIN) level, at the broad‐scale, trophic niche similarity between species was lower in allopatric than in sympatric regions (O_JK_ allopatric = 0.35, O_JK_ sympatric = 0.62). Conversely, at the fine‐scale, within the sympatric region, trophic niche overlap between species was higher in allotopic locations (O_JK_ = 0.56) than in syntopic locations (O_JK_ = 0.37). However, in all the four cases, observed niche overlap between species was higher than 1,000 null models. When measuring trophic niche overlap between species using pairs of locations, we observed the same pattern. At the broad scale, we found higher diet overlap in sympatry than allopatry (O_JK_ sympatric = 0.107 ± 0.056, O_JK_ allopatric = 0.050 ± 0.04; Gausian hurdle model: binomial GLM: z_1,316_ = 4.76, *p* < .05; Gaussian GLM: *t*_1,265_ = 8.26, *p* < .05). In contrast, at the fine‐scale, niche overlap was lower among pairs of syntopic than allotopic locations (O_JK_ syntopic = 0.099 ± 0.065, O_JK_ allotopic = 0.126 ± 0.057; Linear model: *F*_1,73_ = 6.34, *p* = .014; Figure 4b).

### 3.3 Functional diet analysis

**Figure 5:**
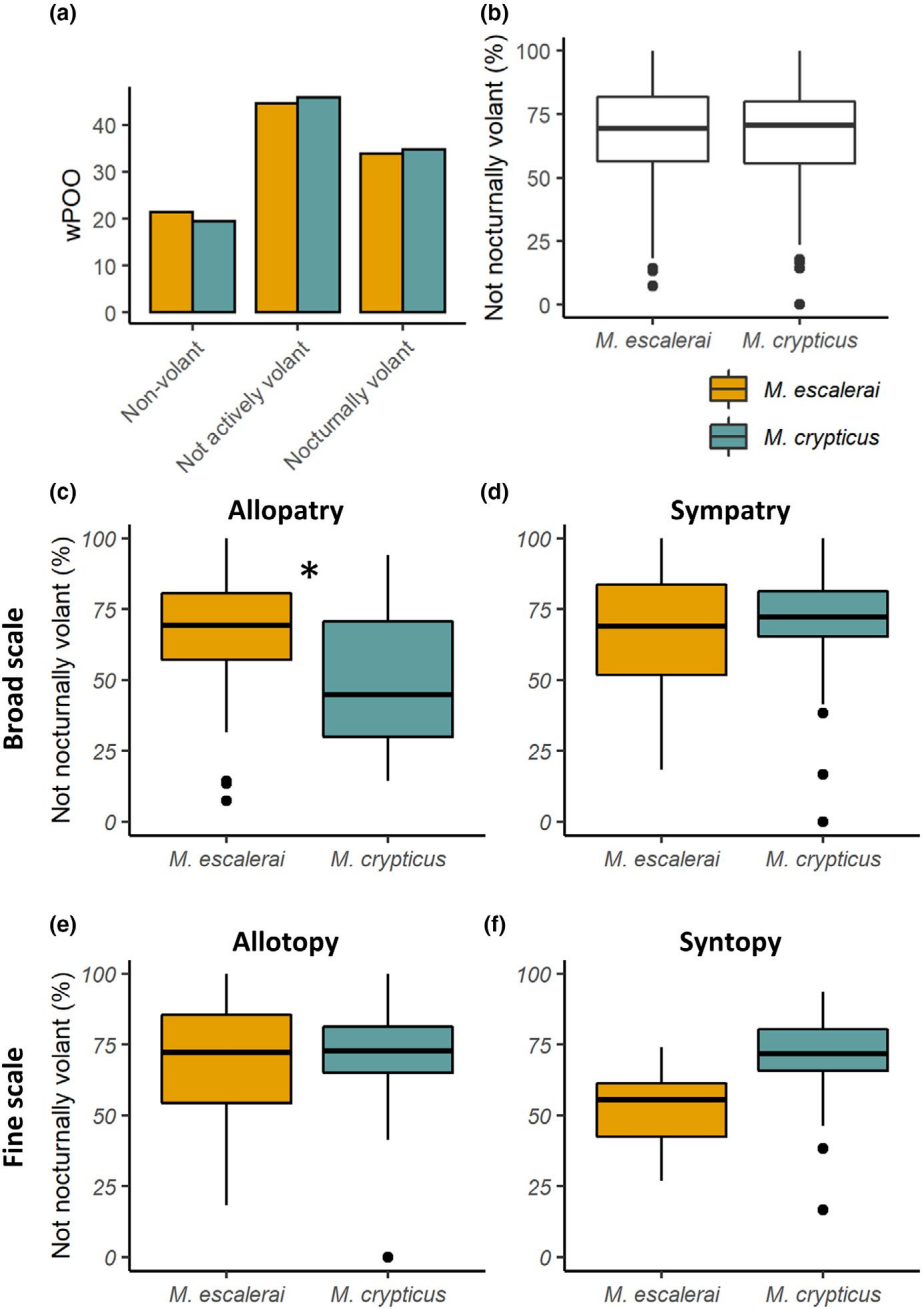
Functional diets of*M. escalerai*and*M. crypticus*depending on the nocturnal flight behavior of the prey species, classified into non‐volant (wingless arthropod groups), not actively volant (able to fly but unlikely to have been captured through aerial hawking), and nocturnally volant (likely to be captured by aerial hawking). Overall proportions of functional categories in the bat diets (a); proportion of not nocturnally volant prey items per dropping sample in*M. escalerai*and*M. crypticus*overall (b), in broad‐scale allopatric versus sympatric regions (c) and in fine‐scale allotopic versus syntopic locations (d). Star denotes significant differences between groups (Linear Model)

Both species had a similar high percentage of non‐volant (*M. escalerai* = 21.4%, *M. crypticus* = 19.5%) and not actively volant (44.6%, 45.8%) prey items in the diet. Only 34.0% and 34.7% of weighted percent of occurrence (wPOO) was composed of arthropods classified as nocturnally volant (Figure 5a). There were no differences in the overall percentage of not nocturnally volant prey taxa (BINs) per sample between bat species (66% ±20%, 66% ±21%, Linear model: *F*_1,217_ < 0.001, *p* = .990; Figure 5b). When analyzing functional diet differences separately in allopatric versus sympatric regions, we found differences between species in allopatric regions, whereby *M. crypticus* consumed lower percentage of prey that were not nocturnally volant (allopatric regions: *M. escalerai* = 66% ±19%, *M. crypticus* = 48% ±25%; *F*_1,98_ = 11.72, *p* < .05; Figure 5c; sympatric regions: 65% ±22%, 71% ±17%; *F*_1,117_ = 2.3, *p* = .13; Figure 5d). At the fine‐scale, there were no differences among bats in allotopic locations (*M. escalerai* = 67% ±22%, *M. crypticus* = 72% ±16%, *F*_1,89_ = 1.325, *p* = .250; Figure 5e) while in syntopic locations the percent of prey that were not nocturnally volant was borderline lower in the diet of *M. escalerai* (52% ±17%) than *M. crypticus* (68% ±18%; *F*_1,26_ = 4.03, *p* = .055; Figure 5f).

### 3.4 Prey consumption relative to availability

The proportion of BINs consumed by the bats that were also present in the sweeping samples of their respective capture sites was highly variable but generally low. In the dropping samples of five sites <10% of the BINs on average also appeared in the sweeping samples, in nine sites 10%–20%, in seven sites 20%–30% and only for two sites >30% of the BINs appeared in the sweeping samples. We retained only the nine sites where at least 20% of the BINs in the bat diet were present in sweeping samples. We detect under‐selection by both species for Diptera, with the distribution of selection values between the 1st and 3rd quartiles falling below zero (1st‐3rd quartiles *M. escalerai*: −16.24 to −1.01, *M. crypticus*: −24.40 to 9.55), and over‐selection by both species for Lepidoptera, with the distribution of selection values between the 1st and 3rd quartiles falling above zero (1st‐3rd quartile *M. escalerai*: 2.04–2.49, *M. crypticus*: 4.30–12.41). In the other two main orders (Areanea and Hemiptera), 1st and 3rd quartiles of selection values overlapped zero (Fig. S5).

### 3.5 Metabarcoding and primer performance

There were compositional differences in the prey orders that each primer recovered. A large proportion of the BINs identified in dropping samples were only recovered by one of the primers. Neuroptera, Orthoptera, and Coleoptera were more frequently recovered by ZBJ, while Plecoptera, Thysanoptera, Dermaptera, and Mantodea were more frequently recovered by ANML (Fig. S6). Figure S7a‐b shows composition for a subset of dropping samples comparing each primer.

In sweeping samples, 7,065 insects were identified morphologically to order level, with an average of 174.3 individuals per sample (range 7–624). Using molecular tools, we recovered 899,853 reads (Table S2), and identified 813 different BIN items. Some of the rarer orders were under‐represented in the molecular analysis. Specifically, Opiliones, Dermaptera, and Archaeognatha appeared in more than 10 sweeping samples each identified morphologically, but were rarely recovered in the molecular approach, despite being present in the reference databases (Fig. S8).

## 4 DISCUSSION

Morphologically almost identical species are likely to compete for resources (Wainwright & Reilly, 1994) and therefore offer a good study system to understand processes that drive species coexistence. If trophic resource partitioning enables species coexistence, trophic similarity is expected to be lower when in sympatry. We find that despite overall very high trophic similarity among the recently separated cryptic bats *M. escalerai* and *M. crypticus*, trophic niche overlap is lower in syntopic relative to allotopic locations. The functional analysis suggests that the subtle trophic shift seen may be driven by differential foraging mode. Our results support niche theory predictions of the role of biotic interactions in driving species assemblages (Schoener, 1974). Trophic resource partitioning was only detected at the fine spatial scale in locations where the two species are syntopic and therefore more likely to compete for resources.

### 4.1 Trophic ecology of *Myotis escalerai* and *Myotis crypticus*

Our results reveal that the two bat species have a broad generalist diet and use gleaning to a high extent. We found a very high similarity in their trophic ecology in terms of both order and prey species composition. Both bat species’ diets are mostly composed of Lepidoptera, Diptera and Araneae, but also include several other prey orders. However, *M. escalerai* consumes a higher percentage of Hemiptera, while *M. crypticu*s Diptera. Functionally, the two bat species consume an equally high proportion of prey items that are not nocturnally volant, which suggests that both bats predominantly glean prey from vegetation.

The trophic ecology of these two recently described bat species is very similar to their cryptic sister‐species *M. nattereri,* which also feeds mostly on Lepidoptera, Diptera and Araneae (Hope et al., 2014; Swift, 1997; Swift & Racey, 2002; Vaughan, 1997) and is known to catch a high proportion of its prey through gleaning (Arlettaz, 1996; Hope et al., 2014; Shiel et al., 1991; Swift, 1997; Swift & Racey, 2002). Similarly to our study, Shiel et al. (1991) estimated that 68% of *M. nattereri's* diet is made up of arthropod families that are not active at night. Although the characteristic row of hairs in the uropatagium border of the *M. nattereri* species complex is thought to be functionally linked with gleaning (Czech et al., 2009) and the presence of more developed hairs in *M. escalerai* is one of the few characteristics which separates these taxa (Juste et al., 2019), we found no difference in the extent of gleaning between the two bat species. This study confirms that the previously identified high proportion of spiders in the diet of *M. nattereri* is common to the rest of the cryptic complex. Spiders are a rare component of the diet of most European bat species, with the exception of *Myotis emarginatus* (Vallejo et al., 2019).

### 4.2 Trophic partitioning across spatial scales

At the prey order level, niche similarity between the two bat species was overall high. However, at the prey species (BIN) level, we detect a signature of trophic shift, whereby diet overlap is lower in syntopic compared to allotopic locations at the fine‐scale. This supports the contribution of trophic partitioning to species coexistence even when overall trophic niche overlap is high, though given the high trophic similarity among the two bat species, other mechanisms of resource partitioning, such as spatial and micro‐habitat partitioning, likely also play an important role. A similar trend is observed at the functional level, whereby the proportion of prey items that are not nocturnally volant is borderline different between bat species only when syntopic. This suggests that the differentiation in diet composition seen at the prey species level when in syntopy may be driven by a shift in foraging strategy (e.g., Krüger et al., 2014), through *M. escalerai* decreasing its extent of gleaning. However, our inference is limited by small sample sizes, which reduced the power of the analysis. At the arthropod order level, we find differences in the use of some arthropod orders among allopatric regions, likely due to differences in arthropod availability between the Mediterranean region, where only *M. escalerai* is found and the Atlantic region, where *M. crypticus* is present.

Several studies have identified trophic niche shifts from allopatry to sympatry, for instance between morphologically similar fish (Gkenas et al., 2019; Schmitt & Coyer, 1983) and reptile species (Huey et al., 1974; Klawinski et al., 1994). However, in bats, previous coexistence studies looking at trophic ecology only focused on sympatric or syntopic populations, and rarely found evidence of trophic resource partitioning. A few exceptions are the gleaning bats *M. nattereri*, *Plecotus auritus,* and *Myotis bechsteini* in a shared roost in central Europe (Andreas et al., 2012a), and evidence of low dietary overlap between co‐occurring *P. auritus* and *Plecotus macrobullaris* (Ashrafi et al., 2011).

The observed trophic shift, albeit subtle, suggests that the two bat species are likely competing for food resources. It has been previously hypothesized that arthropods are abundant and do not constitute a limiting resource for bats (Arlettaz, 1999; Krüger et al., 2014). However, exclusion experiments in both tropical (Kalka et al., 2008) and temperate forests (Böhm et al., 2011) show that bats can control the abundance of arthropods, and therefore, arthropods could be a limiting resource to competitors (Salinas‐Ramos et al., 2020).

Our study does not refute the possibility that other coexistence mechanisms, such as habitat or temporal partitioning (Schoener, 1974), occur among these two species, or the role of environmental variability in facilitating coexistence (Chesson & Warner, 1981). Spatial partitioning is frequently cited as a key mechanism of coexistence in other bat studies (e.g., Arlettaz, 1999; Emrich et al., 2014; Kunz, 1973; Russo et al., 2014). Although in many cases, contrary to our study, spatial partitioning may be driven by slight differences in bat morphology (e.g., Salsamendi et al., 2008, 2012), which would affect their performance in different habitats (Norberg, 1994). However, in our study the two species were caught in the same sampling sites, some of which were forests, where they are known to forage (Juste et al., 2019), suggesting they may share the same foraging sites.

Our finding that trophic partitioning only occurs at the fine spatial scale is consistent with other studies of bats (Peixoto et al., 2018), ants (Albrecht & Gotelli, 2001), parasitoid insects (Harvey et al., 2014) and bobcats (Lewis et al., 2015), showing that interspecific interactions are more important for shaping community structure at fine rather than broad spatial scales. However, this pattern is not universal (e.g., Harmáčková et al., 2019). In our study system, these fine‐scale mechanisms could contribute to enabling broad‐scale range overlap across the north of the Iberian Peninsula because theoretical studies have shown that fine‐scale coexistence mechanisms can prevent competitive effects from scaling‐up and affecting the broad‐scale distributions of species (Godsoe et al., 2015).

### 4.3 Prey consumption relative to availability

The diet of a species is a function of both consumer selection and trophic resource availability within the foraging habitat (Lawlor, 1980). Therefore, considering resource availability allows for a better inference of species trophic preferences. Previous studies comparing bat prey consumption with prey availability pointed to selection of certain prey orders, such as Coleoptera by *Eptesicus fuscus* (Agosta et al., 2003), chironomid flies by *Myotis daubentonii* (Vesterinen et al., 2016) and certain prey traits like moth size by *Barbastella barbastella* (Andreas et al., 2012b). Similarly, *M. nattereri* was found to over‐select arachnids, Opiliones, Coleoptera, and several Diptera families, and under‐select Hemiptera (Swift & Racey, 2002). In this study we detect over‐selection of Lepidoptera and under‐selection of Diptera by both bat species. However, diet selection results should be interpreted with caution. Any arthropod sampling technique is biased toward certain types of arthropods (Cooper & Whitmore, 1990). Our molecular diet analysis results confirm that the two bat species glean prey from the vegetation, and therefore, sweep nets are the appropriate arthropod sampling method to study prey availability. However, the low proportion of BINs in bat diet that appear in sweeping samples does not support the representativeness of our prey availability sampling, likely due to insufficient sampling effort or inadequate coverage of all the areas where the bats forage. This is because bats can use large areas and arthropod communities change depending on habitat type (Lamarre et al., 2016) and vertical stratification (Ulyshen, 2011).

### 4.4 Methodological considerations and study limitations

Primer bias toward certain taxonomic groups is a major issue in metabarcoding studies (Elbrecht et al., 2019). In this study, prey items were frequently recovered by only one of the primers, and differences existed in the recovery of the different arthropod orders. This supports previous studies that suggest that more than one set of primers should be used when the expected diet covers a broader taxonomical spectrum (Alberdi et al., 2018; Aldasoro et al., 2019; Esnaola et al., 2018). The inclusion in this study of a set of samples with known composition based on morphological analysis (albeit only at the order level) gives us some idea of potential biases in the molecular identification. Opiliones, in particular, were morphologically identified in several sweep net samples and are known to be present in the diet of *M. nattereri* (Galan et al., 2018; Swift, 1997; Swift & Racey, 2002), but were absent from the molecularly characterized diets of the two bats. Thus, their absence in this study is likely the result of primer amplification bias.

Because prey development stage cannot be identified using the metabarcoding approach, some of the prey species (BINs) classified as nocturnally volant may correspond to non‐flying larval stages. This could be important in Lepidoptera, and could increase the inferred importance of the gleaned behavior of both species because larval stages are known to be consumed by *M. nattereri* (Hope et al., 2014). Alternatively, prey classified as not active nocturnal fliers can be captured by aerial hawking (e.g., ballooning spiders). More generally, arthropod nocturnal aerial activity is not straightforward to categorize, and therefore, our classification is only tentative. However, potential classification biases are expected to be low and standardized across species because the arthropod orders that are most difficult to categorize due their functional diversity, like Coleoptera, are consumed in similarly low proportion by both bat species. Nevertheless, due to this and low sample sizes in sympatric locations, interpretations of functional prey shift should be considered with caution.

## 5 CONCLUSIONS

In line with niche theory predictions, we show that coexistence among morphologically identical (cryptic) species can be facilitated through fine‐scale mechanisms of resource partitioning, even between species that show high levels of trophic similarity. Our findings that trophic resource partitioning is only detected when bats are syntopic within areas of sympatry suggest that fine‐scale mechanisms of coexistence could have implications for the maintenance of broad‐scale diversity patterns and highlight the importance of using appropriated spatial scales when studying impacts of biotic interactions on community assembly. This is the first study to identify a trophic shift between allotopic and syntopic populations of insectivorous bats, supporting the role of trophic resource partitioning in enabling species co‐occurrence in the same foraging site. It thereby addresses some of the key limitations identified in a recent review of interspecific competition in bats (Salinas‐Ramos et al., 2020). We highlight the importance of using high taxonomic resolution and allopatric populations at meaningful spatial scales for identifying patterns of niche shift, and the utility of using a functional approach that better links mechanistically with species trophic ecology. Understanding mechanisms of coexistence is essential for predicting species vulnerability under climate change because range shifts will result in new community assemblages and competitive interactions (HilleRisLambers et al., 2013). This is particularly relevant in our study system as both species are restricted to the Mediterranean region, where climate change is predicted to be particularly severe (Sala et al., 2000), and both are predicted to experience range shifts and changes in range overlap under climate change (Razgour et al., 2019).

## AUTHOR CONTRIBUTION

**Roberto Novella‐Fernandez:** Data collection (lead); Formal analysis (lead); Investigation (lead); Methodology (equal); Resources (lead); Visualization (lead); Writing‐original draft (lead). **Carlos Ibáñez:** Data curation (equal); Investigation (supporting); Resources (supporting); Supervision (supporting); Writing‐review & editing (equal). **Javier Juste:** Conceptualization (supporting); Data curation (equal); Investigation (supporting); Resources (equal); Supervision (supporting); Writing‐review & editing (equal). **Elizabeth L. Clare:** Formal analysis (supporting); Methodology (supporting); Writing‐review & editing (equal). **C. Patrick Doncaster:** Methodology (supporting); Supervision (supporting); Writing‐review & editing (equal). **Orly Razgour:** Conceptualization (lead); Funding acquisition (lead); Methodology (equal); Supervision (lead); Writing‐review & editing (equal).

## Supporting information

Data file S1

SupInfo

## Data Availability

Metabarcoding sequencing data are deposited in Dryad (https://doi.org/10.5061/dryad.c59zw3r5s). List of arthropod BINs identified in bat fecal samples and their presence in each of the two bat species has been uploaded as Data file S1.
