# Supplementary material for: Trophic resource partitioning drives fine‐scale coexistence in cryptic bat species
Source: Ecol Evol. 2020 Nov 11;10(24):14122–36. doi: 10.1002/ece3.7004 (PMC7771180; doi:10.1002/ece3.7004)
Supplement: Supplementary file 2 — SupInfo [file ECE3-10-14122-s002.pdf]

Supporting Information for:

Trophic resource partitioning drives fine-scale coexistence in cryptic bat species

Roberto Novella-Fernandez[1], Carlos Ibañez[2], Javier Juste[2,3], Elizabeth L. Clare[4], C. Patrick Doncaster[1], Orly Razgour[1, 5]

[1] School of Biological Sciences, University of Southampton, Southampton, UK
[2] Estación Biológica de Doñana (CSIC), Sevilla, Spain.
[3] CIBER Epidemiology and Public Health (CIBERESP), Spain
[4] School of Biological and Chemical Sciences, Queen Mary University of London, UK
[5] Biosciences, University of Exeter, Exeter, UK

Table of Supplementary Tables

Table S1 – Sampling locations and number of dropping samples processed in each location for each bat species (Mes: Myotis escalerai, Mcr: Myotis crypticus). Sampling region and category of the location as regionally sympatric or allopatric or locally sympatric and allopatric. SweepS: Number of sweeping samples analysed. 3
Table S2 – Summary of criteria followed in the functional classification of the BINs recovered according to the likelihood of the bat capturing the arthropod by gleaning or aerial hawking. 0: Non-volant. 1: not actively volant. 2: Nocturnally volant. 4
Table S3 – Number of reads obtained for each sample from each primer (ANML and ZBJ) and replicate (1 and 2). Sample type: D= Dropping sample, S= Sweeping sample. BlankD and BlankSweep: extraction blanks for dropping (D) and sweeping (Sweep) samples. Blankseq: sequencing blank. Failed: If TRUE, sample has been removed under the conservative criteria, due to insufficient number of reads in at least one of the runs. 6
Table S4 – BINs removed from specific samples based on their presence in Extraction blanks. They had either more than 100 reads or less than 10 times more read than in blanks. 13
Table S5 – BINs removed from specific samples and run based on their presence in sequencing blanks. They had either more than 100 reads or less than 10 times more reads than in blanks. 14

Table of Supplementary Figures

Fig. S1 – Flow chart summarising the process followed to to obtain taxonomic prey composition from dropping samples of *M. escalerai* and *M. crypticus*. 22
Fig. S2 – Diet of *Myotis escalerai* (a) and *Myotis crypticus* (b) according to percent frequency of occurrence (POO), weighted percent of occurrence (wPOO) and relative read abundance (RRA). 23
Fig. S3 – Number of BINs/sample of each of the four main Arthropod orders in the diet of *Myotis escalerai* and *Myotis crypticus*. Box represents first and third data quartiles. Whiskers extend from the hinge to the largest value no further than 1.5 * distance between the first and third quartiles. Data beyond the end of the whiskers are plotted individually. Statistically significant differences are noted with a star. 24
Fig. S4 – Number of BINs of each of the four main Arthropod orders in bat samples from sympatric and allopatric locations at the regional scale (a) and at fine scale (b). Statistically significant differences are noted with a star. Boxplot interpretation is detailed in Fig. S3. 25
Fig. S5 – Selection values (bat diet wPOO – Arthropod availability) of *M. escalerai* and *M. crypticus* towards each of the main arthropod orders. Boxplot interpretation is detailed in Fig. S3. 26
Fig. S6 – Number of BINS of each order. Portions coloured depending on the primer from which it was recovered. Red recovered uniquely by ANML, green uniquely by ZBJ, and blue recovered by both. Numbers above columns show number of BINs. 27
Fig. S7 – Prey order composition of a subset of individual dropping samples (RNF 1-21) comparing ANML (left) and ZBJ (right) primers. Number on top of each pie chart shows total BINs recovered. 28
Fig. S8 – Prey order composition of a subset of individual dropping samples (RNF 22-42) comparing ANML (left) and ZBJ (right) primers. Number on top of each pie chart shows total BINs recovered. 29
Fig. S9 – Number of samples in which each order appears in the morphological identification of sweeping samples (total bar height), and portion of those in which the order was present and absent in the subsequent molecular metabarcoding identification. 30

Supplementary Data file S1 – BINs identified in the study, number of samples in which they appear for each bat species and functional classification according to their likelihood of being captured by gleaning or aerial hawking.

Supplementary Tables

Table S1 – Sampling locations and number of dropping samples processed in each location for each bat species (Mes: Myotis escalerai, Mcr: Myotis crypticus). Sampling region and category of the location as regionally sympatric or allopatric or locally sympatric and allopatric. SweepS: Number of sweeping samples analysed.

	Location name	Longitude	Latitude	Sampling Region	Regional sympatry	Local sympatry	Mes	Mcr	SweepS
1	Alquife	-3.11	37.18	Andalusia	Allopatry	NA	2	0	NA
2	Arroyo Tejuelo	-2.57	38.32	Andalusia	Allopatry	NA	3	0	NA
3	Buenamar	-2.78	38.25	Andalusia	Allopatry	NA	1	0	NA
4	Cueva Murcielaguina, Hormos	-2.71	38.22	Andalusia	Allopatry	NA	1	0	NA
5	El Chorro	-3.01	37.87	Andalusia	Allopatry	NA	29	0	NA
6	El Tobazo, Villaescusa de Ebro	-3.83	42.82	Cantabria	Sympatry	Allo	0	1	NA
7	Ermita de Somera	-4.02	42.87	Cantabria	Sympatry	Allo	0	1	NA
8	Fuente de la Garganta	-2.89	37.9	Andalusia	Allopatry	NA	3	0	NA
9	Arroyo Frío o de los Ubios	-2.9	37.94	Andalusia	Allopatry	NA	11	0	2
10	Siles	-2.57	38.32	Andalusia	Allopatry	NA	4	0	1
11	Linarejos	-2.91	37.92	Andalusia	Allopatry	NA	1	0	NA
12	Roblehondo	-2.88	37.95	Andalusia	Allopatry	NA	5	0	1
13	Arroyo de la Canal	-2.96	37.79	Andalusia	Allopatry	NA	6	0	2
14	Mina del Gato	-3.09	37.16	Andalusia	Allopatry	NA	13	0	3
15	Cañada de los Arroyos	-2.98	37.84	Andalusia	Allopatry	NA	1	0	NA
16	Monte Hijedo	-3.93	42.89	Cantabria	Sympatry	Allo	0	2	1
17	Buyezo, Riega de Tornes	-4.5	43.13	Cantabria	Allopatry	NA	0	3	3
18	Rubionzu	-3.81	43.25	Cantabria	Allopatry	NA	0	1	NA
19	Liendo, Manás de la Iseca	-3.36	43.37	Cantabria	Allopatry	NA	0	2	NA
20	Pinar de las nieves	-3.35	43.31	Cantabria	Allopatry	NA	0	1	NA
21	Toberas	-3.37	43.32	Cantabria	Allopatry	NA	0	2	NA
22	Pozos de Valcava	-3.78	43.36	Cantabria	Allopatry	NA	0	1	NA
23	Puente Mercadillo a	-4.22	42.88	Cantabria	Sympatry	Sym	0	7	2
24	Puente Mercadillo p	-4.22	42.88	Cantabria	Sympatry	Sym	1	0	NA
25	Hayedo, Suano	-4.2	42.97	Cantabria	Allopatry	NA	0	1	NA
26	San Martín de Valdelomar	-4.15	42.8	Cantabria	Sympatry	Sym	0	5	1
27	Cuevatón	-4.17	42.81	Cantabria	Sympatry	Sym	1	2	3
28	Coroneles	-4.07	42.82	Cantabria	Sympatry	Allo	0	8	1
29	Ermita de la Velilla	-3.93	42.8	Cantabria	Sympatry	Sym	1	4	1
30	Salcedo	-3.95	42.82	Cantabria	Sympatry	Sym	1	2	1
31	Río Torina	-4.04	43.11	Cantabria	Allopatry	NA	0	1	NA
32	Liébana, San Pedro Bedoya	-4.56	43.18	Cantabria	Allopatry	NA	0	3	2
33	Ginebret	2.7	42.28	Catalonia	Sympatry	NA	4	5	1
34	El Rasillo	-2.72	42.2	La Rioja	Sympatry	Allo	0	3	2
35	Ermita de Lollano	-2.66	42.15	La Rioja	Sympatry	Allo	0	17	1
36	Cueva del Moro, Valgañón	-3.08	42.32	La Rioja	Sympatry	Sym	2	1	1
37	Calamantio	-2.93	42.19	La Rioja	Sympatry	Allo	0	2	NA
38	Valvanera	-2.87	42.23	La Rioja	Sympatry	Allo	0	1	NA
39	Cueva Cuatro puertas	-2.25	42.2	La Rioja	Sympatry	Allo	3	0	1
40	Cueva Cerraúco	-2.74	42.18	La Rioja	Sympatry	Allo	0	4	1
41	Túnel Arnedillo	-2.2	42.2	La Rioja	Sympatry	Allo	8	0	2
42	Dehesa de Villoslada	-2.68	42.12	La Rioja	Sympatry	Allo	0	6	1
43	Cueva Azagra	-1.87	42.28	Navarra	Sympatry	Allo	15	0	NA
44	Cueva del Agua, Villaciervos	-2.66	41.78	Castilla la Mancha	Sympatry	Allo	20	0	1
45	Parador Nacional	-2.96	37.9	Andalusia	Allopatry	NA	1	0	NA
46	Peña Olivar	-2.58	38.37	Andalusia	Allopatry	NA	1	0	NA
47	Quejigal, Villacantiz	-4.19	43	Cantabria	Allopatry	NA	0	2	NA
48	Regato Troncos, la Matilla	-3.94	42.82	Cantabria	Sympatry	Sym	0	1	NA
49	Vega de Villafufre	-3.85	43.26	Cantabria	Allopatry	NA	0	1	NA

Table S2 – Summary of criteria followed in the functional classification of the BINs recovered according to the likelihood of the bat capturing the arthropod by gleaning or aerial hawking. 0: Non-volant. 1: not actively volant. 2: Nocturnally volant.

Order	Number of BINs	Functional groups	Criteria used	References
**Araneae**	140	0	Morphological	
**Archaeognatha**	1	0	Morphological	
**Blattodea**	7	0, 1	Morphological	
**Coleoptera**	87	1,2	Literature	(Haack, Keena, & Eyre, 2017) (Cerambycidae)(Nalepa, 2013) (Coccinelidae)
**Dermaptera**	2	0	Morphological	
**Diptera**	423	1, 2	Taxonomist (Jorge Mederos), Literature	(Oosterbroek, 2006)
**Ephemeroptera**	6	2	Literature	
**Hemiptera**	96	1	Literature	(Waloff & Bakker, 1963)
**Hymenoptera**	68	1, 2	Literature	(Broad, 2005; Lozan, Belokobylskij, Achterberg, & Monaghan, 2010; Mazon, Bordera, & Rodríguez-Berrío, 2009; Quicke, 2014; Short, Schmidt, & Steinbauer, 2006)(Ichneumonoidea, (Tilgner, 2004) (Tenthredinidae)
**Isopoda**	2	0	Morphological	
**Lepidoptera**	560	2	Literature	
**Mantodea**	1	1	own observations	
**Mecoptera**	1	2	Literature	
**Neuroptera**	19	2	Literature	Vaset al 1999
**Odonata**	5	1	Literature	(Corbet, 1999)
**Orthoptera**	36	1	Literature	
**Plecoptera**	1	2	Literature	
**Thysanoptera**	2	0	Morphological	
**Trichoptera**	6	2	Literature	

Broad, G. R. (2005). Keys for the identification of British and Irish nocturnal Ichneumonidae.

Corbet, P. S. (1999). *Dragonflies: Behaviour and Ecology of Odonata* (Revised ed). Harley Books.

Haack, R. A., Keena, M. A., & Eyre, D. (2017). Life history and population dynamics of cerambycids. *Cerambycidae of the World: Biology and Pest Management*, 71–103. doi:10.1201/b21851

Lozan, A. I., Belokobylskij, S., Achterberg, C. van Van, & Monaghan, M. T. (2010). Diversity and Distribution of Braconidae, a Family of Parasitoid Wasps in the Central European Peatbogs of South Bohemia, Czech Republic. *Journal of Insect Science*, *10*(16), 1–21. doi:10.1673/031.010.1601

Mazon, M., Bordera, S., & Rodríguez-Berrío, A. (2009). Diurnal flight activity of Ichneumonidae (Insecta: Hymenoptera) in Cabañeros National Park (Spain). *Journal of Natural History*, *43*(21–22), 1291–1304. doi:10.1080/00222930902878966

Nalepa, C. A. (2013). Coccinellidae captured in blacklight traps: Seasonal and diel pattern of the dominant species Harmonia axyridis (Coleoptera: Coccinellidae). *European Journal of Entomology*, *110*(4), 593–597. doi:10.14411/eje.2013.080

Oosterbroek, P. (2006). *The European Families of the Diptera*. *The European Families of the Diptera*. KNNV Publishing. doi:10.1163/9789004278066

Quicke, D. L. J. (2014). *The Braconid and Ichneumonid Parasitoid Wasps*. Chichester, UK: John Wiley & Sons, Ltd. doi:10.1002/9781118907085

Short, M., Schmidt, S., & Steinbauer, M. (2006). A key to some Australian genera of large nocturnal Ichneumonidae (Hymenoptera), including flight periodicities and influence of moon phase on light trap catches. *Australian Entomologist, The*, *33*(1), 49–55.

Tilgner, E. H. (2004). *Encyclopedia of Entomology*. (J. L. Capinera, Ed.), *Encyclopedia of Insects*. doi:10.1016/B978-0-12-374144-8.00203-4

Waloff, N., & Bakker, K. (1963). The Flight Activity of Miridae (Heteroptera) Living on Broom, Sarothamnus scoparius (L.). *Journal of Animal Ecology*, *32*(3), 461–480.

Table S3 – Number of reads obtained for each sample from each primer (ANML and ZBJ) and replicate (1 and 2). Sample type: D= Dropping sample, S= Sweeping sample. BlankD and BlankSweep: extraction blanks for dropping (D) and sweeping (Sweep) samples. Blankseq: sequencing blank. Failed: If TRUE, sample has been removed under the conservative criteria, due to insufficient number of reads in at least one of the runs.

Sample name	ANML_1	ANML_2	ZBJ_1	ZBJ_2	Sample type	Failed
**RNF_1**	3	30	839	2077	D	TRUE
**RNF_2**	4129	749	3271	7860	D	.
**RNF_3**	6015	8336	4615	8843	D	.
**RNF_4**	2779	2262	3967	3587	D	.
**RNF_5**	542	252	576	1310	D	.
**RNF_6**	5586	10536	5546	6788	D	.
**RNF_7**	441	610	1658	2735	D	.
**RNF_8**	3426	1385	3454	3254	D	.
**RNF_9**	761	310	2533	2198	D	.
**RNF_10**	2098	2015	3010	3960	D	.
**RNF_11**	571	614	484	447	D	.
**RNF_12**	265	159	546	376	D	.
**RNF_13**	2528	1075	2539	604	D	.
**RNF_14**	6233	7633	4613	4177	D	.
**RNF_15**	149	77	30	37	D	TRUE
**RNF_16**	7072	3610	2349	2680	D	.
**RNF_17**	1993	1934	216	251	D	.
**RNF_18**	2121	4254	526	419	D	.
**RNF_19**	2086	3701	4638	4492	D	.
**RNF_20**	6443	7418	3284	3187	D	.
**RNF_21**	3352	7350	1961	3072	D	.
**RNF_22**	2	772	39	81	D	TRUE
**RNF_23**	3700	1544	1546	320	D	.
**RNF_24**	1150	4350	2603	3363	D	.
**RNF_25**	649	2122	29	512	D	TRUE
**RNF_26**	669	247	2691	2537	D	.
**RNF_27**	3303	4512	4815	6308	D	.
**RNF_28**	6148	7204	1727	3799	D	.
**RNF_29**	144	668	3089	7763	D	.
**RNF_30**	45	25	147	1150	D	TRUE
**RNF_31**	372	330	1591	1802	D	.
**RNF_32**	338	136	1837	1287	D	.
**RNF_33**	6	7	967	888	D	TRUE
**RNF_34**	2929	3541	52	9	D	TRUE
**RNF_35**	3039	9730	4951	5396	D	.
**RNF_36**	6277	6511	4911	7008	D	.
**RNF_37**	6088	14238	4779	6216	D	.
**RNF_38**	5	9957	7127	6530	D	TRUE

**RNF_39**	6167	8827	2018	2368	D	.
**RNF_40**	6519	6599	7520	6833	D	.
**RNF_41**	3444	6308	4627	5045	D	.
**RNF_42**	3738	6604	1598	1822	D	.
**RNF_43**	3436	13103	1163	181	D	.
**RNF_44**	4311	9291	541	5673	D	.
**RNF_45**	932	91	38	62	D	TRUE
**RNF_46**	3227	7602	3995	4363	D	.
**RNF_47**	6441	9061	6231	7498	D	.
**RNF_48**	1	3307	5372	6126	D	TRUE
**RNF_49**	2109	3973	4135	4434	D	.
**RNF_50**	3139	3421	7682	6700	D	.
**RNF_52**	46	1270	308	3337	D	TRUE
**RNF_53**	205	37	1054	3325	D	TRUE
**RNF_54**	1776	4670	1215	1306	D	.
**RNF_55**	110	75	66	72	D	TRUE
**RNF_56**	1360	1226	1336	1032	D	.
**RNF_57**	2260	1974	393	745	D	.
**RNF_58**	4156	5322	1124	1120	D	.
**RNF_59**	3	6320	975	1320	D	TRUE
**RNF_60**	5098	2525	366	578	D	.
**RNF_61**	1248	1579	1191	1391	D	.
**RNF_62**	5	2	1	8	D	TRUE
**RNF_63**	0	9	576	3	D	TRUE
**RNF_64**	2426	3131	5563	6426	D	.
**RNF_65**	1592	54	3583	5333	D	TRUE
**RNF_66**	1	467	62	14	D	TRUE
**RNF_67**	4008	3366	5413	5077	D	.
**RNF_68**	4480	8082	5730	6941	D	.
**RNF_69**	2330	3157	4818	5430	D	.
**RNF_70**	3477	6658	4769	4402	D	.
**RNF_71**	4111	7231	2528	922	D	.
**RNF_72**	1247	1629	2404	2133	D	.
**RNF_73**	7623	540	2515	3077	D	.
**RNF_74**	4321	5151	4381	6201	D	.
**RNF_75**	3140	5082	5570	6323	D	.
**RNF_76**	3150	4352	5146	6508	D	.
**RNF_77**	1256	1349	6153	5925	D	.
**RNF_78**	625	2246	2658	1740	D	.
**RNF_79**	3134	3759	2076	851	D	.
**RNF_80**	1911	3627	4292	3688	D	.
**RNF_81**	2447	3028	1306	1337	D	.
**RNF_82**	2820	2844	896	1001	D	.
**RNF_83**	3114	715	8495	7349	D	.
**RNF_84**	4330	3322	3637	6789	D	.

**RNF_85**	4789	5204	6422	7623	D	.
**RNF_86**	2954	9595	5067	6202	D	.
**RNF_87**	5940	11539	5340	6157	D	.
**RNF_88**	4735	10223	1815	3362	D	.
**RNF_89**	5501	9866	3752	4250	D	.
**RNF_90**	88	2448	81	297	D	TRUE
**RNF_91**	4266	8944	3216	3091	D	.
**RNF_92**	6646	4014	5294	6902	D	.
**RNF_93**	4813	8490	4773	4622	D	.
**RNF_94**	5237	4735	4001	5707	D	.
**RNF_95**	6347	8863	5118	6249	D	.
**RNF_96**	7575	7644	3536	5274	D	.
**RNF_97**	4823	7053	2823	2572	D	.
**RNF_98**	6786	7938	4842	2629	D	.
**RNF_99**	2985	1398	4005	4316	D	.
**RNF_100**	0	364	6	1	D	TRUE
**RNF_101**	7102	8656	6250	5290	D	.
**RNF_102**	111	21	1840	1607	D	TRUE
**RNF_103**	3844	3541	5300	5460	D	.
**RNF_104**	3150	6490	447	386	D	.
**RNF_105**	9	4	157	55	D	TRUE
**RNF_106**	1165	0	3323	3841	D	TRUE
**RNF_107**	5289	7522	8277	6929	D	.
**RNF_108**	3217	1652	4594	2726	D	.
**RNF_109**	80	6234	60	2099	D	TRUE
**RNF_110**	1716	2300	4433	5770	D	.
**RNF_111**	1886	2110	2617	3050	D	.
**RNF_112**	2537	2155	1457	1490	D	.
**RNF_113**	1508	939	3303	3006	D	.
**RNF_114**	6116	7359	5986	6927	D	.
**RNF_115**	5192	6602	4217	4112	D	.
**RNF_116**	694	597	2225	1768	D	.
**RNF_117**	4657	4713	4673	5374	D	.
**RNF_118**	5395	5967	2967	2711	D	.
**RNF_119**	3979	4633	4394	5807	D	.
**RNF_120**	4379	5066	6049	6878	D	.
**RNF_121**	7091	4851	4227	4442	D	.
**RNF_122**	98	66	2168	2321	D	TRUE
**RNF_123**	2242	3263	4219	5631	D	.
**RNF_124**	3157	3062	2815	5928	D	.
**RNF_125**	362	333	1324	1296	D	.
**RNF_126**	2527	2035	2281	1814	D	.
**RNF_127**	4780	6045	3036	3034	D	.
**RNF_128**	5264	7624	3298	3899	D	.
**RNF_129**	2984	3260	1875	1593	D	.

**RNF_131**	4252	3201	7604	7903	D	.
**RNF_133**	1427	1039	3688	3910	D	.
**RNF_134**	3824	990	7214	6999	D	.
**RNF_135**	604	800	3536	959	D	.
**RNF_136**	5365	858	1739	1494	D	.
**RNF_138**	3060	4052	4669	6847	D	.
**RNF_139**	3444	4457	6446	5079	D	.
**RNF_140**	3197	507	5524	5978	D	.
**RNF_141**	3683	4173	4753	1072	D	.
**RNF_142**	2798	1278	107	2610	D	.
**RNF_143**	7502	8895	7143	9080	D	.
**RNF_144**	193	142	6769	4863	D	.
**RNF_145**	1	371	162	3884	D	TRUE
**RNF_146**	4123	6550	1961	8827	D	.
**RNF_147**	962	654	3436	3621	D	.
**RNF_148**	2806	3660	3483	3877	D	.
**RNF_149**	1173	927	1277	1269	D	.
**RNF_150**	2483	1417	6663	6024	D	.
**RNF_151**	1103	1253	18	17	D	TRUE
**RNF_152**	54	53	229	176	D	TRUE
**RNF_153**	5144	4410	5626	6397	D	.
**RNF_154**	3261	4193	4023	4331	D	.
**RNF_155**	1871	2347	1059	2661	D	.
**RNF_156**	718	397	567	599	D	.
**RNF_157**	4676	1109	4751	4233	D	.
**RNF_158**	580	625	2520	2480	D	.
**RNF_159**	4122	4938	5894	5099	D	.
**RNF_160**	843	4150	5843	7612	D	.
**RNF_161**	6199	6701	6677	7871	D	.
**RNF_162**	1458	1633	8001	7235	D	.
**RNF_163**	1480	836	4547	4094	D	.
**RNF_164**	5441	6215	7241	7267	D	.
**RNF_165**	4082	4721	975	1243	D	.
**RNF_166**	5439	5815	7199	7689	D	.
**RNF_167**	7025	7161	6153	7877	D	.
**RNF_168**	6703	7241	8372	8283	D	.
**RNF_169**	5960	8014	7334	8111	D	.
**RNF_170**	4916	3021	6141	7413	D	.
**RNF_171**	3935	4037	3470	5026	D	.
**RNF_172**	4617	4919	1375	6815	D	.
**RNF_173**	4845	5264	2605	3099	D	.
**RNF_174**	3541	2691	4243	2401	D	.
**RNF_175**	11	39	81	2	D	TRUE
**RNF_176**	982	152	8	3	D	TRUE
**RNF_177**	2743	1905	2015	3542	D	.

**RNF_178**	4644	1184	1919	3845	D	.
**RNF_179**	659	352	356	714	D	.
**RNF_180**	355	241	4	5	D	TRUE
**RNF_181**	719	219	513	32	D	TRUE
**RNF_182**	2906	684	25	36	D	TRUE
**RNF_183**	103	60	1287	1052	D	TRUE
**RNF_184**	1086	5	278	291	D	TRUE
**RNF_185**	350	554	101	277	D	.
**RNF_186**	120	128	8	5	D	TRUE
**RNF_187**	86	24	64	24	D	TRUE
**RNF_189**	1857	1441	1069	1159	D	.
**RNF_191**	1395	492	5304	5253	D	.
**RNF_192**	44	37	154	157	D	TRUE
**RNF_193**	1523	2329	810	3436	D	.
**RNF_194**	1917	1364	1371	1799	D	.
**RNF_195**	141	90	10	19	D	TRUE
**RNF_197**	4713	3298	651	1028	D	.
**RNF_199**	11	6	2	1	D	TRUE
**RNF_201**	3384	4436	2877	3498	D	.
**RNF_202**	5097	6577	43	6268	D	TRUE
**RNF_203**	2168	1852	2666	3115	D	.
**RNF_204**	2601	1099	5408	4882	D	.
**RNF_206**	20	9	90	564	D	TRUE
**RNF_207**	10	19	3	620	D	TRUE
**RNF_208**	3263	3977	1348	1644	D	.
**RNF_209**	189	6629	4384	6337	D	.
**RNF_210**	24	115	12	4	D	TRUE
**RNF_211**	6015	7813	1755	3155	D	.
**RNF_212**	31	4	2	105	D	TRUE
**RNF_213**	4380	5181	3236	2747	D	.
**RNF_214**	6248	7061	4687	5508	D	.
**RNF_215**	4209	5465	2486	2231	D	.
**RNF_216**	5070	5091	4007	4107	D	.
**RNF_217**	1091	2224	2362	4847	D	.
**RNF_218**	1902	1994	159	247	D	.
**RNF_219**	113	2	8	7168	D	TRUE
**RNF_220**	1881	1906	524	556	D	.
**RNF_221**	799	667	1212	2991	D	.
**RNF_223**	85	28	3640	3845	D	TRUE
**RNF_224**	6	9	84	433	D	TRUE
**RNF_226**	1422	1743	2376	2776	D	.
**RNF_229**	822	618	589	652	D	.
**RNF_231**	26	8	2750	2391	D	TRUE
**RNF_234**	38	8	33	5	D	TRUE
**RNF_235**	1003	972	3419	3627	D	.

**RNF_236**	21	7	4039	4344	D	TRUE
**RNF_239**	925	1302	369	1402	D	.
**RNF_240**	7792	8727	6179	7988	D	.
**RNF_241**	5326	6637	6102	7075	D	.
**RNF_242**	2850	2317	251	267	D	.
**RNF_243**	40	13	42	32	D	TRUE
**RNF_244**	851	1743	414	1961	D	.
**RNF_248**	5276	5072	5790	7816	D	.
**RNF_249**	4544	5122	3474	3411	D	.
**RNF_251**	7569	7149	7588	8956	D	.
**RNF_B10**	5	205	22	2	BlankD	.
**RNF_B14**	1	5	222	9	BlankD	.
**RNF_B2**	36	3	0	101	BlankD	.
**RNF_B4**	12	9	3	4	BlankD	.
**RNF_BS1**	28	1	0	59	BlankSweep	.
**RNF_S1A**	2721	3433	3616	4713	S	.
**RNF_S1B**	3892	5309	4309	5364	S	.
**RNF_S2**	3683	3624	3234	3333	S	.
**RNF_S5**	2182	3408	2575	3053	S	.
**RNF_S6A**	4661	6410	4070	5612	S	.
**RNF_S6B**	5825	10345	5076	6571	S	.
**RNF_S7A**	427	303	782	1369	S	.
**RNF_S7B**	5410	6218	5150	5301	S	.
**RNF_S7C**	1605	1957	1033	1536	S	.
**RNF_S9A**	6007	7438	4362	5368	S	.
**RNF_S9B**	4957	7397	3659	5258	S	.
**RNF_S10A**	4903	7393	1687	3377	S	.
**RNF_S10B**	3008	5563	3720	5096	S	.
**RNF_S11**	3160	4694	3595	4348	S	.
**RNF_S12A**	7278	9584	6244	7521	S	.
**RNF_S12B**	4373	4934	3280	3804	S	.
**RNF_S13A**	5244	7284	5526	5081	S	.
**RNF_S13A(2)**	2998	3454	5259	6543	S	.
**RNF_S13B**	3591	6380	5316	5434	S	.
**RNF_S13B(2)**	7771	7322	6704	7391	S	.
**RNF_S14**	2422	2350	3481	4608	S	.
**RNF_S17**	3510	3084	2626	3666	S	.
**RNF_S18A**	6063	4411	4986	6718	S	.
**RNF_S18B**	6169	7288	5871	6400	S	.
**RNF_S19A**	7090	9155	7223	8311	S	.
**RNF_S19B**	2419	4835	6949	8085	S	.
**RNF_S20A**	2577	2162	3005	3604	S	.
**RNF_S20B**	7805	5498	5901	6610	S	.
**RNF_S21**	3829	3563	3740	3473	S	.
**RNF_S25A**	6585	4753	4692	6217	S	.

**RNF_S25B**	10220	6346	6604	6839	S	.
**RNF_S25C**	6625	6353	6693	6334	S	.
**RNF_S26A**	6577	5389	8583	7922	S	.
**RNF_S26B**	5974	4564	7523	8999	S	.
**RNF_S26C**	8655	7406	7441	8409	S	.
**RNF_S31A**	4310	5690	4514	4762	S	.
**RNF_S31B**	5959	4798	6715	5267	S	.
**RNF_S33**	6876	7165	4873	6014	S	.
**RNF_S34A**	6414	4449	6819	7131	S	.
**RNF_S34B**	6350	6948	6825	7253	S	.
**RNF_S34C**	7882	3835	7449	10340	S	.
**RNF_S35**	4734	4591	1695	3005	S	.
**RNF_S36**	5852	5626	4809	4297	S	.
**RNF_Seq Blanc1**	3	1	3	19	Blankseq	.
**RNF_Seq Blanc2**	2	1	4	8	Blankseq	.
**RNF_Seq Blanc3**	24	4	22	8	Blankseq	.
**RNF_Seq Blanc4**	10	8	3	162	Blankseq	.
**RNF_Seq Blanc5**	6	1	4	125	Blankseq	.

Table S4 – BINs removed from specific samples based on their presence in Extraction blanks. These smaples had less than 10 times more reads than in blanks.

BIN	Sample	Order	Family	Genus	Species
**BOLD:AAB9618**	RNF_175	Lepidoptera	Crambidae	Pyrausta	Pyrausta despicata
**BOLD:AAB9618**	RNF_240	Lepidoptera	Crambidae	Pyrausta	Pyrausta despicata
**BOLD:AAD0642**	RNF_240	Diptera	Muscidae	Helina	Helina duplicata, Helina reversio
**BOLD:AAD0642**	RNF_249	Diptera	Muscidae	Helina	Helina duplicata, Helina reversio
**BOLD:AAH3467**	RNF_249	Hymenoptera	Braconidae	Homolobus	
**BOLD:AAH3467**	RNF_249	Hymenoptera	Braconidae	Homolobus	
**BOLD:AAP2403**	RNF_147	Araneae	Philodromidae	Philodromus	Philodromus dispar
**BOLD:AAP2403**	RNF_147	Araneae	Philodromidae	Philodromus	Philodromus dispar
**BOLD:AAP2403**	RNF_147	Araneae	Philodromidae	Philodromus	Philodromus dispar
**BOLD:AAP2403**	RNF_147	Araneae	Philodromidae	Philodromus	Philodromus dispar
**BOLD:AAP2403**	RNF_148	Araneae	Philodromidae	Philodromus	Philodromus dispar
**BOLD:AAP2403**	RNF_175	Araneae	Philodromidae	Philodromus	Philodromus dispar
**BOLD:AAP2403**	RNF_176	Araneae	Philodromidae	Philodromus	Philodromus dispar
**BOLD:AAP2403**	RNF_178	Araneae	Philodromidae	Philodromus	Philodromus dispar
**BOLD:AAP2403**	RNF_239	Araneae	Philodromidae	Philodromus	Philodromus dispar
**BOLD:AAP2403**	RNF_240	Araneae	Philodromidae	Philodromus	Philodromus dispar
**BOLD:ACB4574**	RNF_147	Diptera	Lauxaniidae	Calliopum	Calliopum aeneum
**BOLD:ACB4575**	RNF_242	Diptera	Lauxaniidae	Calliopum	Calliopum elisae
**BOLD:ACD2919**	RNF_240	Diptera	Anthomyiidae	Delia	Delia penicillosa, Delia penicillaris
**BOLD:ACL7274**	RNF_129	Blattodea	Ectobiidae	Ectobius	Ectobius pallidus
**BOLD:ACL7274**	RNF_129	Blattodea	Ectobiidae	Ectobius	Ectobius pallidus

Table S5 – BINs removed from specific samples and run based on their presence in sequencing blanks. These samples had less than 10 times more reads than in blanks.

BIN	Sample	Run	Order	Genus	Species
**BOLD:AAB9618**	RNF_9	ZBJ_2	Lepidoptera	Pyrausta	Pyrausta despicata
**BOLD:AAB9618**	RNF_16	ZBJ_2	Lepidoptera	Pyrausta	Pyrausta despicata
**BOLD:AAB9618**	RNF_25	ZBJ_2	Lepidoptera	Pyrausta	Pyrausta despicata
**BOLD:AAB9618**	RNF_26	ZBJ_2	Lepidoptera	Pyrausta	Pyrausta despicata
**BOLD:AAB9618**	RNF_29	ZBJ_2	Lepidoptera	Pyrausta	Pyrausta despicata
**BOLD:AAB9618**	RNF_49	ZBJ_2	Lepidoptera	Pyrausta	Pyrausta despicata
**BOLD:AAB9618**	RNF_57	ZBJ_2	Lepidoptera	Pyrausta	Pyrausta despicata
**BOLD:AAB9618**	RNF_81	ZBJ_2	Lepidoptera	Pyrausta	Pyrausta despicata
**BOLD:AAB9618**	RNF_82	ZBJ_2	Lepidoptera	Pyrausta	Pyrausta despicata
**BOLD:AAB9618**	RNF_113	ZBJ_2	Lepidoptera	Pyrausta	Pyrausta despicata
**BOLD:AAB9618**	RNF_162	ZBJ_2	Lepidoptera	Pyrausta	Pyrausta despicata
**BOLD:AAB9618**	RNF_169	ZBJ_2	Lepidoptera	Pyrausta	Pyrausta despicata
**BOLD:AAB9618**	RNF_197	ZBJ_2	Lepidoptera	Pyrausta	Pyrausta despicata
**BOLD:AAB9618**	RNF_204	ZBJ_2	Lepidoptera	Pyrausta	Pyrausta despicata
**BOLD:AAB9618**	RNF_207	ZBJ_2	Lepidoptera	Pyrausta	Pyrausta despicata
**BOLD:AAC1503**	RNF_16	ZBJ_2	Trichoptera	Limnephilus	Limnephilus graecus, Limnephilus lunatus
**BOLD:AAC1503**	RNF_57	ZBJ_2	Trichoptera	Limnephilus	Limnephilus graecus, Limnephilus lunatus
**BOLD:AAC1503**	RNF_169	ZBJ_2	Trichoptera	Limnephilus	Limnephilus graecus, Limnephilus lunatus
**BOLD:AAC1503**	RNF_207	ZBJ_2	Trichoptera	Limnephilus	Limnephilus graecus, Limnephilus lunatus
**BOLD:AAC2028**	RNF_109	ZBJ_1	Lepidoptera	Udea	Udea prunalis
**BOLD:AAC5906**	RNF_24	ZBJ_1	Lepidoptera	Endotricha	Endotricha flammealis
**BOLD:AAC5906**	RNF_144	ZBJ_1	Lepidoptera	Endotricha	Endotricha flammealis
**BOLD:AAC5906**	RNF_159	ZBJ_1	Lepidoptera	Endotricha	Endotricha flammealis
**BOLD:AAC5906**	RNF_187	ZBJ_1	Lepidoptera	Endotricha	Endotricha flammealis
**BOLD:AAC5906**	RNF_209	ZBJ_1	Lepidoptera	Endotricha	Endotricha flammealis
**BOLD:AAC5906**	RNF_248	ZBJ_1	Lepidoptera	Endotricha	Endotricha flammealis
**BOLD:AAE6869**	RNF_121	ANML_2	Lepidoptera	Agriphila	Agriphila geniculea, Agriphila tolli, Agriphila sp.
**BOLD:AAF5224**	RNF_40	ZBJ_2	Araneae	Linyphia	Linyphia tenuipalpis
**BOLD:AAF5224**	RNF_68	ZBJ_2	Araneae	Linyphia	Linyphia tenuipalpis
**BOLD:AAF5224**	RNF_81	ZBJ_2	Araneae	Linyphia	Linyphia tenuipalpis
**BOLD:AAF5224**	RNF_118	ZBJ_2	Araneae	Linyphia	Linyphia tenuipalpis
**BOLD:AAF5224**	RNF_167	ZBJ_2	Araneae	Linyphia	Linyphia tenuipalpis
**BOLD:AAF5224**	RNF_171	ZBJ_2	Araneae	Linyphia	Linyphia tenuipalpis
**BOLD:AAF5224**	RNF_207	ZBJ_2	Araneae	Linyphia	Linyphia tenuipalpis
**BOLD:AAF5224**	RNF_214	ZBJ_2	Araneae	Linyphia	Linyphia tenuipalpis
**BOLD:AAF5224**	RNF_231	ZBJ_2	Araneae	Linyphia	Linyphia tenuipalpis
**BOLD:AAF5224**	RNF_241	ZBJ_2	Araneae	Linyphia	Linyphia tenuipalpis

**BOLD:AAF5224**	RNF_248	ZBJ_2	Araneae	Linyphia	Linyphia tenuipalpis
**BOLD:AAF5224**	RNF_251	ZBJ_2	Araneae	Linyphia	Linyphia tenuipalpis
**BOLD:AAF9059**	RNF_9	ZBJ_2	Diptera	Rhipidia	Rhipidia maculata
**BOLD:AAF9059**	RNF_16	ZBJ_2	Diptera	Rhipidia	Rhipidia maculata
**BOLD:AAF9059**	RNF_25	ZBJ_2	Diptera	Rhipidia	Rhipidia maculata
**BOLD:AAF9059**	RNF_26	ZBJ_2	Diptera	Rhipidia	Rhipidia maculata
**BOLD:AAF9059**	RNF_49	ZBJ_2	Diptera	Rhipidia	Rhipidia maculata
**BOLD:AAF9059**	RNF_57	ZBJ_2	Diptera	Rhipidia	Rhipidia maculata
**BOLD:AAF9059**	RNF_81	ZBJ_2	Diptera	Rhipidia	Rhipidia maculata
**BOLD:AAF9059**	RNF_113	ZBJ_2	Diptera	Rhipidia	Rhipidia maculata
**BOLD:AAF9059**	RNF_157	ZBJ_2	Diptera	Rhipidia	Rhipidia maculata
**BOLD:AAF9059**	RNF_159	ZBJ_2	Diptera	Rhipidia	Rhipidia maculata
**BOLD:AAF9059**	RNF_162	ZBJ_2	Diptera	Rhipidia	Rhipidia maculata
**BOLD:AAF9059**	RNF_169	ZBJ_2	Diptera	Rhipidia	Rhipidia maculata
**BOLD:AAF9059**	RNF_197	ZBJ_2	Diptera	Rhipidia	Rhipidia maculata
**BOLD:AAF9059**	RNF_207	ZBJ_2	Diptera	Rhipidia	Rhipidia maculata
**BOLD:AAF9059**	RNF_240	ZBJ_2	Diptera	Rhipidia	Rhipidia maculata
**BOLD:AAF9059**	RNF_251	ZBJ_2	Diptera	Rhipidia	Rhipidia maculata
**BOLD:AAG2511**	RNF_6	ZBJ_2	Diptera	Delia	Delia platura
**BOLD:AAG2511**	RNF_9	ZBJ_2	Diptera	Delia	Delia platura
**BOLD:AAG2511**	RNF_10	ZBJ_2	Diptera	Delia	Delia platura
**BOLD:AAG2511**	RNF_16	ZBJ_2	Diptera	Delia	Delia platura
**BOLD:AAG2511**	RNF_26	ZBJ_2	Diptera	Delia	Delia platura
**BOLD:AAG2511**	RNF_33	ZBJ_2	Diptera	Delia	Delia platura
**BOLD:AAG2511**	RNF_46	ZBJ_2	Diptera	Delia	Delia platura
**BOLD:AAG2511**	RNF_49	ZBJ_2	Diptera	Delia	Delia platura
**BOLD:AAG2511**	RNF_53	ZBJ_2	Diptera	Delia	Delia platura
**BOLD:AAG2511**	RNF_57	ZBJ_2	Diptera	Delia	Delia platura
**BOLD:AAG2511**	RNF_77	ZBJ_2	Diptera	Delia	Delia platura
**BOLD:AAG2511**	RNF_87	ZBJ_2	Diptera	Delia	Delia platura
**BOLD:AAG2511**	RNF_95	ZBJ_2	Diptera	Delia	Delia platura
**BOLD:AAG2511**	RNF_115	ZBJ_2	Diptera	Delia	Delia platura
**BOLD:AAG2511**	RNF_116	ZBJ_2	Diptera	Delia	Delia platura
**BOLD:AAG2511**	RNF_121	ZBJ_2	Diptera	Delia	Delia platura
**BOLD:AAG2511**	RNF_124	ZBJ_2	Diptera	Delia	Delia platura
**BOLD:AAG2511**	RNF_126	ZBJ_2	Diptera	Delia	Delia platura
**BOLD:AAG2511**	RNF_127	ZBJ_2	Diptera	Delia	Delia platura
**BOLD:AAG2511**	RNF_128	ZBJ_2	Diptera	Delia	Delia platura
**BOLD:AAG2511**	RNF_131	ZBJ_2	Diptera	Delia	Delia platura
**BOLD:AAG2511**	RNF_149	ZBJ_2	Diptera	Delia	Delia platura
**BOLD:AAG2511**	RNF_161	ZBJ_2	Diptera	Delia	Delia platura
**BOLD:AAG2511**	RNF_162	ZBJ_2	Diptera	Delia	Delia platura
**BOLD:AAG2511**	RNF_169	ZBJ_2	Diptera	Delia	Delia platura
**BOLD:AAG2511**	RNF_178	ZBJ_2	Diptera	Delia	Delia platura
**BOLD:AAG2511**	RNF_194	ZBJ_2	Diptera	Delia	Delia platura

**BOLD:AAG2511**	RNF_195	ZBJ_2	Diptera	Delia	Delia platura
**BOLD:AAG2511**	RNF_207	ZBJ_2	Diptera	Delia	Delia platura
**BOLD:AAG2511**	RNF_215	ZBJ_2	Diptera	Delia	Delia platura
**BOLD:AAG2511**	RNF_224	ZBJ_2	Diptera	Delia	Delia platura
**BOLD:AAG2511**	RNF_229	ZBJ_2	Diptera	Delia	Delia platura
**BOLD:AAH4103**	RNF_16	ZBJ_2	Isopoda	Philoscia	Philoscia muscorum
**BOLD:AAH4103**	RNF_57	ZBJ_2	Isopoda	Philoscia	Philoscia muscorum
**BOLD:AAH4103**	RNF_81	ZBJ_2	Isopoda	Philoscia	Philoscia muscorum
**BOLD:AAH4103**	RNF_105	ZBJ_2	Isopoda	Philoscia	Philoscia muscorum
**BOLD:AAH4103**	RNF_113	ZBJ_2	Isopoda	Philoscia	Philoscia muscorum
**BOLD:AAH4103**	RNF_168	ZBJ_2	Isopoda	Philoscia	Philoscia muscorum
**BOLD:AAH4103**	RNF_219	ZBJ_2	Isopoda	Philoscia	Philoscia muscorum
**BOLD:AAJ4117**	RNF_161	ZBJ_2	Araneae	Clubiona	Clubiona terrestris
**BOLD:AAJ4117**	RNF_167	ZBJ_2	Araneae	Clubiona	Clubiona terrestris
**BOLD:AAJ4117**	RNF_207	ZBJ_2	Araneae	Clubiona	Clubiona terrestris
**BOLD:AAJ4117**	RNF_208	ZBJ_2	Araneae	Clubiona	Clubiona terrestris
**BOLD:AAJ4117**	RNF_226	ZBJ_2	Araneae	Clubiona	Clubiona terrestris
**BOLD:AAJ4117**	RNF_240	ZBJ_2	Araneae	Clubiona	Clubiona terrestris
**BOLD:AAJ4117**	RNF_251	ZBJ_2	Araneae	Clubiona	Clubiona terrestris
**BOLD:AAO3252**	RNF_16	ZBJ_2	Araneae	Anyphaena	Anyphaena accentuata
**BOLD:AAO3252**	RNF_25	ZBJ_2	Araneae	Anyphaena	Anyphaena accentuata
**BOLD:AAO3252**	RNF_57	ZBJ_2	Araneae	Anyphaena	Anyphaena accentuata
**BOLD:AAO3252**	RNF_69	ZBJ_2	Araneae	Anyphaena	Anyphaena accentuata
**BOLD:AAO3252**	RNF_71	ZBJ_2	Araneae	Anyphaena	Anyphaena accentuata
**BOLD:AAO3252**	RNF_81	ZBJ_2	Araneae	Anyphaena	Anyphaena accentuata
**BOLD:AAO3252**	RNF_105	ZBJ_2	Araneae	Anyphaena	Anyphaena accentuata
**BOLD:AAO3252**	RNF_113	ZBJ_2	Araneae	Anyphaena	Anyphaena accentuata
**BOLD:AAO3252**	RNF_159	ZBJ_2	Araneae	Anyphaena	Anyphaena accentuata
**BOLD:AAO3252**	RNF_163	ZBJ_2	Araneae	Anyphaena	Anyphaena accentuata
**BOLD:AAO3252**	RNF_164	ZBJ_2	Araneae	Anyphaena	Anyphaena accentuata
**BOLD:AAO3252**	RNF_165	ZBJ_2	Araneae	Anyphaena	Anyphaena accentuata
**BOLD:AAO3252**	RNF_251	ZBJ_2	Araneae	Anyphaena	Anyphaena accentuata
**BOLD:AAO4492**	RNF_149	ANML_1	Araneae	Episinus	Episinus maculipes
**BOLD:AAO4492**	RNF_167	ANML_1	Araneae	Episinus	Episinus maculipes
**BOLD:AAO4492**	RNF_169	ANML_1	Araneae	Episinus	Episinus maculipes
**BOLD:AAP2403**	RNF_5	ANML_1	Araneae	Philodromus	Philodromus dispar
**BOLD:AAP2403**	RNF_9	ANML_1	Araneae	Philodromus	Philodromus dispar
**BOLD:AAP2403**	RNF_11	ANML_1	Araneae	Philodromus	Philodromus dispar
**BOLD:AAP2403**	RNF_12	ANML_1	Araneae	Philodromus	Philodromus dispar
**BOLD:AAP2403**	RNF_16	ANML_1	Araneae	Philodromus	Philodromus dispar
**BOLD:AAP2403**	RNF_35	ANML_1	Araneae	Philodromus	Philodromus dispar
**BOLD:AAP2403**	RNF_49	ANML_1	Araneae	Philodromus	Philodromus dispar
**BOLD:AAP2403**	RNF_52	ANML_1	Araneae	Philodromus	Philodromus dispar
**BOLD:AAP2403**	RNF_53	ANML_1	Araneae	Philodromus	Philodromus dispar
**BOLD:AAP2403**	RNF_58	ANML_1	Araneae	Philodromus	Philodromus dispar

**BOLD:AAP2403**	RNF_65	ANML_1	Araneae	Philodromus	Philodromus dispar
**BOLD:AAP2403**	RNF_71	ANML_1	Araneae	Philodromus	Philodromus dispar
**BOLD:AAP2403**	RNF_83	ANML_1	Araneae	Philodromus	Philodromus dispar
**BOLD:AAP2403**	RNF_94	ANML_1	Araneae	Philodromus	Philodromus dispar
**BOLD:AAP2403**	RNF_103	ANML_1	Araneae	Philodromus	Philodromus dispar
**BOLD:AAP2403**	RNF_106	ANML_1	Araneae	Philodromus	Philodromus dispar
**BOLD:AAP2403**	RNF_113	ANML_1	Araneae	Philodromus	Philodromus dispar
**BOLD:AAP2403**	RNF_133	ANML_1	Araneae	Philodromus	Philodromus dispar
**BOLD:AAP2403**	RNF_134	ANML_1	Araneae	Philodromus	Philodromus dispar
**BOLD:AAP2403**	RNF_141	ANML_1	Araneae	Philodromus	Philodromus dispar
**BOLD:AAP2403**	RNF_143	ANML_1	Araneae	Philodromus	Philodromus dispar
**BOLD:AAP2403**	RNF_148	ANML_1	Araneae	Philodromus	Philodromus dispar
**BOLD:AAP2403**	RNF_164	ANML_1	Araneae	Philodromus	Philodromus dispar
**BOLD:AAP2403**	RNF_174	ANML_1	Araneae	Philodromus	Philodromus dispar
**BOLD:AAP2403**	RNF_187	ANML_1	Araneae	Philodromus	Philodromus dispar
**BOLD:AAP2403**	RNF_191	ANML_1	Araneae	Philodromus	Philodromus dispar
**BOLD:AAP2403**	RNF_204	ANML_1	Araneae	Philodromus	Philodromus dispar
**BOLD:AAP2403**	RNF_208	ANML_1	Araneae	Philodromus	Philodromus dispar
**BOLD:AAP2403**	RNF_210	ANML_1	Araneae	Philodromus	Philodromus dispar
**BOLD:AAP2403**	RNF_211	ANML_1	Araneae	Philodromus	Philodromus dispar
**BOLD:AAP2403**	RNF_218	ANML_1	Araneae	Philodromus	Philodromus dispar
**BOLD:AAP2403**	RNF_219	ANML_1	Araneae	Philodromus	Philodromus dispar
**BOLD:AAP2403**	RNF_243	ANML_1	Araneae	Philodromus	Philodromus dispar
**BOLD:AAP2403**	RNF_249	ANML_1	Araneae	Philodromus	Philodromus dispar
**BOLD:AAP2825**	RNF_50	ZBJ_2	Diptera	Pollenia	Pollenia angustigena, Pollenia rudis
**BOLD:AAP2825**	RNF_53	ZBJ_2	Diptera	Pollenia	Pollenia angustigena, Pollenia rudis
**BOLD:AAP2825**	RNF_57	ZBJ_2	Diptera	Pollenia	Pollenia angustigena, Pollenia rudis
**BOLD:AAP2825**	RNF_82	ZBJ_2	Diptera	Pollenia	Pollenia angustigena, Pollenia rudis
**BOLD:AAP2825**	RNF_114	ZBJ_2	Diptera	Pollenia	Pollenia angustigena, Pollenia rudis
**BOLD:AAP2825**	RNF_116	ZBJ_2	Diptera	Pollenia	Pollenia angustigena, Pollenia rudis
**BOLD:AAP2825**	RNF_118	ZBJ_2	Diptera	Pollenia	Pollenia angustigena, Pollenia rudis
**BOLD:AAP2825**	RNF_120	ZBJ_2	Diptera	Pollenia	Pollenia angustigena, Pollenia rudis
**BOLD:AAP2825**	RNF_127	ZBJ_2	Diptera	Pollenia	Pollenia angustigena, Pollenia rudis
**BOLD:AAP2825**	RNF_169	ZBJ_2	Diptera	Pollenia	Pollenia angustigena, Pollenia rudis
**BOLD:AAP2825**	RNF_207	ZBJ_2	Diptera	Pollenia	Pollenia angustigena, Pollenia rudis
**BOLD:AAU3916**	RNF_207	ZBJ_2	Lepidoptera	Telphusa	Telphusa cistiflorella
**BOLD:ABA6492**	RNF_16	ZBJ_2	Diptera	Hylemya	Hylemya vagans, Hylemya nigrimana
**BOLD:ABA6492**	RNF_25	ZBJ_2	Diptera	Hylemya	Hylemya vagans, Hylemya nigrimana
**BOLD:ABA6492**	RNF_57	ZBJ_2	Diptera	Hylemya	Hylemya vagans, Hylemya nigrimana
**BOLD:ABA6492**	RNF_81	ZBJ_2	Diptera	Hylemya	Hylemya vagans, Hylemya nigrimana
**BOLD:ABA6492**	RNF_169	ZBJ_2	Diptera	Hylemya	Hylemya vagans, Hylemya nigrimana

**BOLD:ABA6492**	RNF_207	ZBJ_2	Diptera	Hylemya	Hylemya vagans, Hylemya nigrimana
**BOLD:ABA6492**	RNF_208	ZBJ_2	Diptera	Hylemya	Hylemya vagans, Hylemya nigrimana
**BOLD:ABA6492**	RNF_215	ZBJ_2	Diptera	Hylemya	Hylemya vagans, Hylemya nigrimana
**BOLD:ABA6492**	RNF_216	ZBJ_2	Diptera	Hylemya	Hylemya vagans, Hylemya nigrimana
**BOLD:ABA6492**	RNF_224	ZBJ_2	Diptera	Hylemya	Hylemya vagans, Hylemya nigrimana
**BOLD:ABA6492**	RNF_226	ZBJ_2	Diptera	Hylemya	Hylemya vagans, Hylemya nigrimana
**BOLD:ABA6492**	RNF_231	ZBJ_2	Diptera	Hylemya	Hylemya vagans, Hylemya nigrimana
**BOLD:ABA6492**	RNF_240	ZBJ_2	Diptera	Hylemya	Hylemya vagans, Hylemya nigrimana
**BOLD:ABA6492**	RNF_241	ZBJ_2	Diptera	Hylemya	Hylemya vagans, Hylemya nigrimana
**BOLD:ABA6492**	RNF_251	ZBJ_2	Diptera	Hylemya	Hylemya vagans, Hylemya nigrimana
**BOLD:ABX4957**	RNF_16	ZBJ_2	Lepidoptera	Cyclophora	Cyclophora ruficiliaria
**BOLD:ABX4957**	RNF_57	ZBJ_2	Lepidoptera	Cyclophora	Cyclophora ruficiliaria
**BOLD:ABX4957**	RNF_81	ZBJ_2	Lepidoptera	Cyclophora	Cyclophora ruficiliaria
**BOLD:ABX4957**	RNF_91	ZBJ_2	Lepidoptera	Cyclophora	Cyclophora ruficiliaria
**BOLD:ABX4957**	RNF_113	ZBJ_2	Lepidoptera	Cyclophora	Cyclophora ruficiliaria
**BOLD:ABX4957**	RNF_215	ZBJ_2	Lepidoptera	Cyclophora	Cyclophora ruficiliaria
**BOLD:ABX4957**	RNF_224	ZBJ_2	Lepidoptera	Cyclophora	Cyclophora ruficiliaria
**BOLD:ABX4957**	RNF_231	ZBJ_2	Lepidoptera	Cyclophora	Cyclophora ruficiliaria
**BOLD:ABX4957**	RNF_240	ZBJ_2	Lepidoptera	Cyclophora	Cyclophora ruficiliaria
**BOLD:ABX4957**	RNF_251	ZBJ_2	Lepidoptera	Cyclophora	Cyclophora ruficiliaria
**BOLD:ACB4574**	RNF_57	ZBJ_2	Diptera	Calliopum	Calliopum aeneum
**BOLD:ACB4574**	RNF_69	ZBJ_2	Diptera	Calliopum	Calliopum aeneum
**BOLD:ACB4574**	RNF_82	ZBJ_2	Diptera	Calliopum	Calliopum aeneum
**BOLD:ACB4574**	RNF_87	ZBJ_2	Diptera	Calliopum	Calliopum aeneum
**BOLD:ACB4574**	RNF_95	ZBJ_2	Diptera	Calliopum	Calliopum aeneum
**BOLD:ACB4574**	RNF_102	ZBJ_2	Diptera	Calliopum	Calliopum aeneum
**BOLD:ACB4574**	RNF_123	ZBJ_2	Diptera	Calliopum	Calliopum aeneum
**BOLD:ACB4574**	RNF_126	ZBJ_2	Diptera	Calliopum	Calliopum aeneum
**BOLD:ACB4574**	RNF_128	ZBJ_2	Diptera	Calliopum	Calliopum aeneum
**BOLD:ACB4574**	RNF_131	ZBJ_2	Diptera	Calliopum	Calliopum aeneum
**BOLD:ACB4574**	RNF_134	ZBJ_2	Diptera	Calliopum	Calliopum aeneum
**BOLD:ACB4574**	RNF_136	ZBJ_2	Diptera	Calliopum	Calliopum aeneum
**BOLD:ACB4574**	RNF_161	ZBJ_2	Diptera	Calliopum	Calliopum aeneum
**BOLD:ACB4574**	RNF_207	ZBJ_2	Diptera	Calliopum	Calliopum aeneum
**BOLD:ACB4574**	RNF_209	ZBJ_2	Diptera	Calliopum	Calliopum aeneum
**BOLD:ACD9629**	RNF_81	ZBJ_2	Diptera	Stevenia	Stevenia deceptoria, Stevenia umbratica
**BOLD:ACD9629**	RNF_97	ZBJ_2	Diptera	Stevenia	Stevenia deceptoria, Stevenia umbratica

**BOLD:ACD9629**	RNF_162	ZBJ_2	Diptera	Stevenia	Stevenia deceptoria, Stevenia umbratica
**BOLD:ACD9629**	RNF_197	ZBJ_2	Diptera	Stevenia	Stevenia deceptoria, Stevenia umbratica
**BOLD:ACD9629**	RNF_240	ZBJ_2	Diptera	Stevenia	Stevenia deceptoria, Stevenia umbratica
**BOLD:ACD9629**	RNF_251	ZBJ_2	Diptera	Stevenia	Stevenia deceptoria, Stevenia umbratica
**BOLD:ACF3089**	RNF_5	ANML_2	Lepidoptera	Agrotis	Agrotis crassa, Agrotis bigramma
**BOLD:ACF3089**	RNF_14	ANML_2	Lepidoptera	Agrotis	Agrotis crassa, Agrotis bigramma
**BOLD:ACF3089**	RNF_25	ZBJ_2	Lepidoptera	Agrotis	Agrotis crassa, Agrotis bigramma
**BOLD:ACF3089**	RNF_26	ZBJ_2	Lepidoptera	Agrotis	Agrotis crassa, Agrotis bigramma
**BOLD:ACF3089**	RNF_40	ZBJ_2	Lepidoptera	Agrotis	Agrotis crassa, Agrotis bigramma
**BOLD:ACF3089**	RNF_45	ANML_2	Lepidoptera	Agrotis	Agrotis crassa, Agrotis bigramma
**BOLD:ACF3089**	RNF_76	ANML_2	Lepidoptera	Agrotis	Agrotis crassa, Agrotis bigramma
**BOLD:ACF3089**	RNF_82	ZBJ_2	Lepidoptera	Agrotis	Agrotis crassa, Agrotis bigramma
**BOLD:ACF3089**	RNF_95	ANML_2	Lepidoptera	Agrotis	Agrotis crassa, Agrotis bigramma
**BOLD:ACF3089**	RNF_113	ZBJ_2	Lepidoptera	Agrotis	Agrotis crassa, Agrotis bigramma
**BOLD:ACF3089**	RNF_114	ZBJ_2	Lepidoptera	Agrotis	Agrotis crassa, Agrotis bigramma
**BOLD:ACF3089**	RNF_139	ANML_2	Lepidoptera	Agrotis	Agrotis crassa, Agrotis bigramma
**BOLD:ACF3089**	RNF_158	ANML_2	Lepidoptera	Agrotis	Agrotis crassa, Agrotis bigramma
**BOLD:ACF3089**	RNF_169	ZBJ_2	Lepidoptera	Agrotis	Agrotis crassa, Agrotis bigramma
**BOLD:ACF3089**	RNF_169	ANML_2	Lepidoptera	Agrotis	Agrotis crassa, Agrotis bigramma
**BOLD:ACF3089**	RNF_179	ANML_2	Lepidoptera	Agrotis	Agrotis crassa, Agrotis bigramma
**BOLD:ACF3089**	RNF_197	ZBJ_2	Lepidoptera	Agrotis	Agrotis crassa, Agrotis bigramma
**BOLD:ACF3089**	RNF_207	ZBJ_2	Lepidoptera	Agrotis	Agrotis crassa, Agrotis bigramma
**BOLD:ACF3089**	RNF_240	ZBJ_2	Lepidoptera	Agrotis	Agrotis crassa, Agrotis bigramma
**BOLD:ACJ2905**	RNF_74	ZBJ_1	Diptera	Diastata	
**BOLD:ACL7274**	RNF_12	ANML_1	Blattodea	Ectobius	Ectobius pallidus
**BOLD:ACL7274**	RNF_23	ZBJ_2	Blattodea	Ectobius	Ectobius pallidus
**BOLD:ACL7274**	RNF_25	ZBJ_2	Blattodea	Ectobius	Ectobius pallidus
**BOLD:ACL7274**	RNF_28	ZBJ_2	Blattodea	Ectobius	Ectobius pallidus
**BOLD:ACL7274**	RNF_29	ZBJ_2	Blattodea	Ectobius	Ectobius pallidus
**BOLD:ACL7274**	RNF_42	ANML_1	Blattodea	Ectobius	Ectobius pallidus
**BOLD:ACL7274**	RNF_53	ZBJ_2	Blattodea	Ectobius	Ectobius pallidus
**BOLD:ACL7274**	RNF_54	ANML_1	Blattodea	Ectobius	Ectobius pallidus
**BOLD:ACL7274**	RNF_57	ZBJ_2	Blattodea	Ectobius	Ectobius pallidus
**BOLD:ACL7274**	RNF_59	ZBJ_2	Blattodea	Ectobius	Ectobius pallidus
**BOLD:ACL7274**	RNF_70	ANML_1	Blattodea	Ectobius	Ectobius pallidus
**BOLD:ACL7274**	RNF_77	ZBJ_2	Blattodea	Ectobius	Ectobius pallidus
**BOLD:ACL7274**	RNF_78	ANML_1	Blattodea	Ectobius	Ectobius pallidus
**BOLD:ACL7274**	RNF_81	ZBJ_2	Blattodea	Ectobius	Ectobius pallidus
**BOLD:ACL7274**	RNF_82	ANML_1	Blattodea	Ectobius	Ectobius pallidus
**BOLD:ACL7274**	RNF_82	ZBJ_2	Blattodea	Ectobius	Ectobius pallidus
**BOLD:ACL7274**	RNF_95	ZBJ_2	Blattodea	Ectobius	Ectobius pallidus
**BOLD:ACL7274**	RNF_103	ANML_1	Blattodea	Ectobius	Ectobius pallidus

**BOLD:ACL7274**	RNF_104	ANML_1	Blattodea	Ectobius	Ectobius pallidus
**BOLD:ACL7274**	RNF_104	ZBJ_2	Blattodea	Ectobius	Ectobius pallidus
**BOLD:ACL7274**	RNF_105	ZBJ_2	Blattodea	Ectobius	Ectobius pallidus
**BOLD:ACL7274**	RNF_120	ANML_1	Blattodea	Ectobius	Ectobius pallidus
**BOLD:ACL7274**	RNF_128	ANML_1	Blattodea	Ectobius	Ectobius pallidus
**BOLD:ACL7274**	RNF_129	ZBJ_2	Blattodea	Ectobius	Ectobius pallidus
**BOLD:ACL7274**	RNF_133	ZBJ_2	Blattodea	Ectobius	Ectobius pallidus
**BOLD:ACL7274**	RNF_140	ZBJ_2	Blattodea	Ectobius	Ectobius pallidus
**BOLD:ACL7274**	RNF_141	ANML_1	Blattodea	Ectobius	Ectobius pallidus
**BOLD:ACL7274**	RNF_147	ANML_1	Blattodea	Ectobius	Ectobius pallidus
**BOLD:ACL7274**	RNF_160	ANML_1	Blattodea	Ectobius	Ectobius pallidus
**BOLD:ACL7274**	RNF_169	ANML_1	Blattodea	Ectobius	Ectobius pallidus
**BOLD:ACL7274**	RNF_171	ANML_1	Blattodea	Ectobius	Ectobius pallidus
**BOLD:ACL7274**	RNF_202	ANML_1	Blattodea	Ectobius	Ectobius pallidus
**BOLD:ACL7274**	RNF_215	ZBJ_2	Blattodea	Ectobius	Ectobius pallidus
**BOLD:ACL7274**	RNF_219	ANML_1	Blattodea	Ectobius	Ectobius pallidus
**BOLD:ACL7274**	RNF_226	ZBJ_2	Blattodea	Ectobius	Ectobius pallidus
**BOLD:ACL7274**	RNF_229	ANML_1	Blattodea	Ectobius	Ectobius pallidus
**BOLD:ACL7274**	RNF_231	ANML_1	Blattodea	Ectobius	Ectobius pallidus
**BOLD:ACL7274**	RNF_240	ZBJ_2	Blattodea	Ectobius	Ectobius pallidus
**BOLD:ACL7274**	RNF_251	ANML_1	Blattodea	Ectobius	Ectobius pallidus
**BOLD:ACL7274**	RNF_251	ZBJ_2	Blattodea	Ectobius	Ectobius pallidus
**BOLD:ACR4033**	RNF_3	ZBJ_2	Diptera	Coenosia	Coenosia albicornis
**BOLD:ACR4033**	RNF_56	ZBJ_2	Diptera	Coenosia	Coenosia albicornis
**BOLD:ACR4033**	RNF_115	ZBJ_2	Diptera	Coenosia	Coenosia albicornis
**BOLD:ACR4033**	RNF_126	ZBJ_2	Diptera	Coenosia	Coenosia albicornis
**BOLD:ACR4033**	RNF_194	ZBJ_2	Diptera	Coenosia	Coenosia albicornis
**BOLD:ACR4033**	RNF_201	ZBJ_2	Diptera	Coenosia	Coenosia albicornis
**BOLD:ACR4033**	RNF_223	ZBJ_2	Diptera	Coenosia	Coenosia albicornis
**BOLD:ACT0928**	RNF_249	ZBJ_2	Hemiptera	Aphrophora	Aphrophora corticea
**BOLD:ADB5574**	RNF_25	ZBJ_2	Diptera		
**BOLD:ADB5574**	RNF_57	ZBJ_2	Diptera		
**BOLD:ADB5574**	RNF_169	ZBJ_2	Diptera		
**BOLD:ADB5574**	RNF_207	ZBJ_2	Diptera		
**BOLD:ADC8699**	RNF_9	ZBJ_2	Hymenoptera	Campoletis	
**BOLD:ADC8699**	RNF_16	ZBJ_2	Hymenoptera	Campoletis	
**BOLD:ADC8699**	RNF_25	ZBJ_2	Hymenoptera	Campoletis	
**BOLD:ADC8699**	RNF_26	ZBJ_2	Hymenoptera	Campoletis	
**BOLD:ADC8699**	RNF_49	ZBJ_2	Hymenoptera	Campoletis	
**BOLD:ADC8699**	RNF_57	ZBJ_2	Hymenoptera	Campoletis	
**BOLD:ADC8699**	RNF_81	ZBJ_2	Hymenoptera	Campoletis	
**BOLD:ADC8699**	RNF_86	ZBJ_1	Hymenoptera	Campoletis	
**BOLD:ADC8699**	RNF_113	ZBJ_2	Hymenoptera	Campoletis	
**BOLD:ADC8699**	RNF_162	ZBJ_2	Hymenoptera	Campoletis	
**BOLD:ADC8699**	RNF_169	ZBJ_2	Hymenoptera	Campoletis	

**BOLD:ADC8699**	RNF_175	ZBJ_1	Hymenoptera	Campoletis	
**BOLD:ADC8699**	RNF_197	ZBJ_2	Hymenoptera	Campoletis	
**BOLD:ADC8699**	RNF_207	ZBJ_2	Hymenoptera	Campoletis	
**BOLD:ADC8699**	RNF_240	ZBJ_2	Hymenoptera	Campoletis	
**BOLD:ADC8699**	RNF_251	ZBJ_2	Hymenoptera	Campoletis	
**BOLD:ADG2771**	RNF_26	ANML_1	Coleoptera	Stagetus	Stagetus ferreri
**BOLD:ADG2771**	RNF_206	ANML_1	Coleoptera	Stagetus	Stagetus ferreri
**BOLD:ADG2771**	RNF_207	ANML_1	Coleoptera	Stagetus	Stagetus ferreri
**BOLD:ADG2771**	RNF_223	ANML_1	Coleoptera	Stagetus	Stagetus ferreri

Supplementary Figures

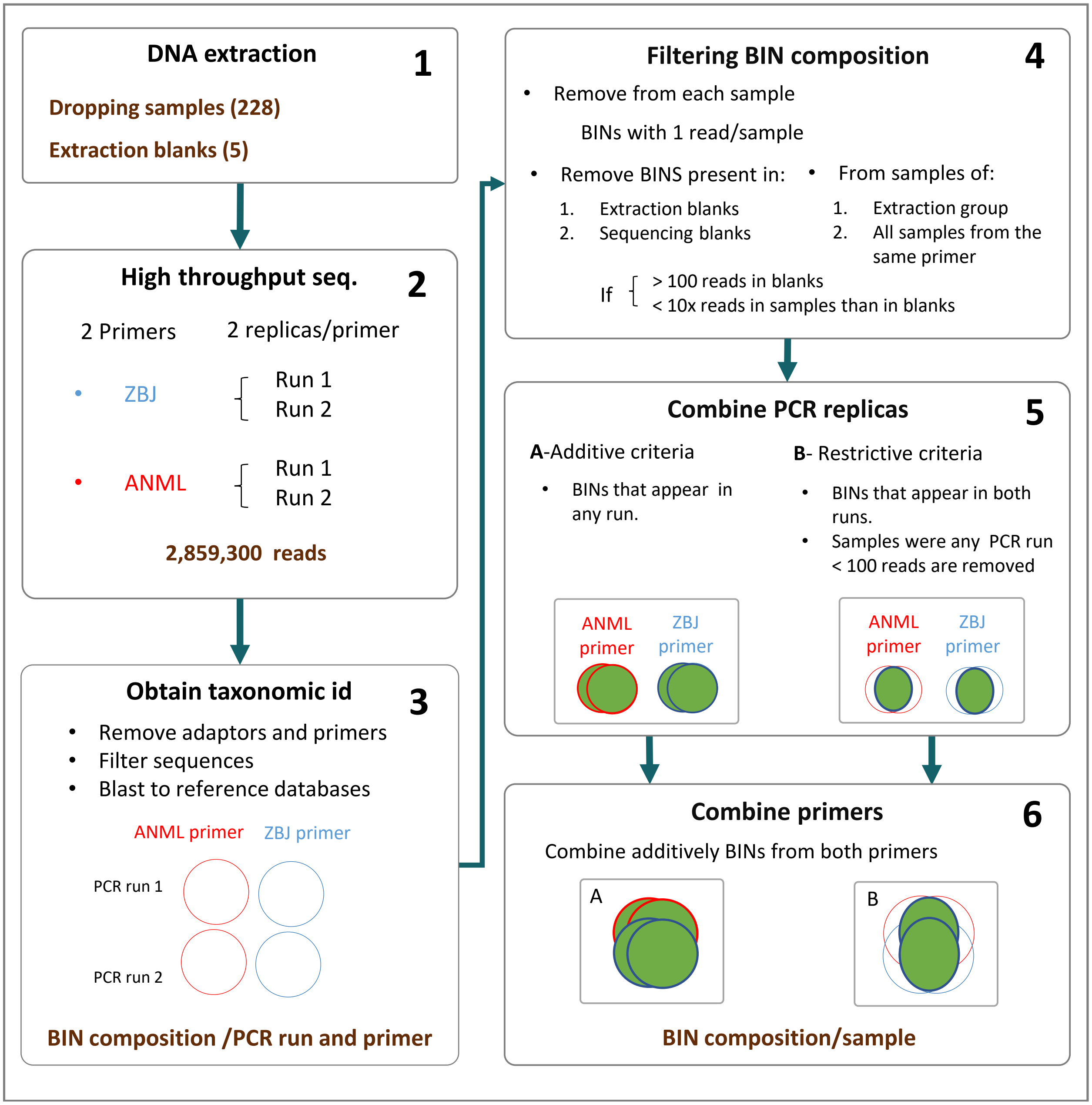

Fig. S1 – Flow chart summarising the process followed to to obtain taxonomic prey composition from dropping samples of *M. escalerai* and *M. crypticus*.

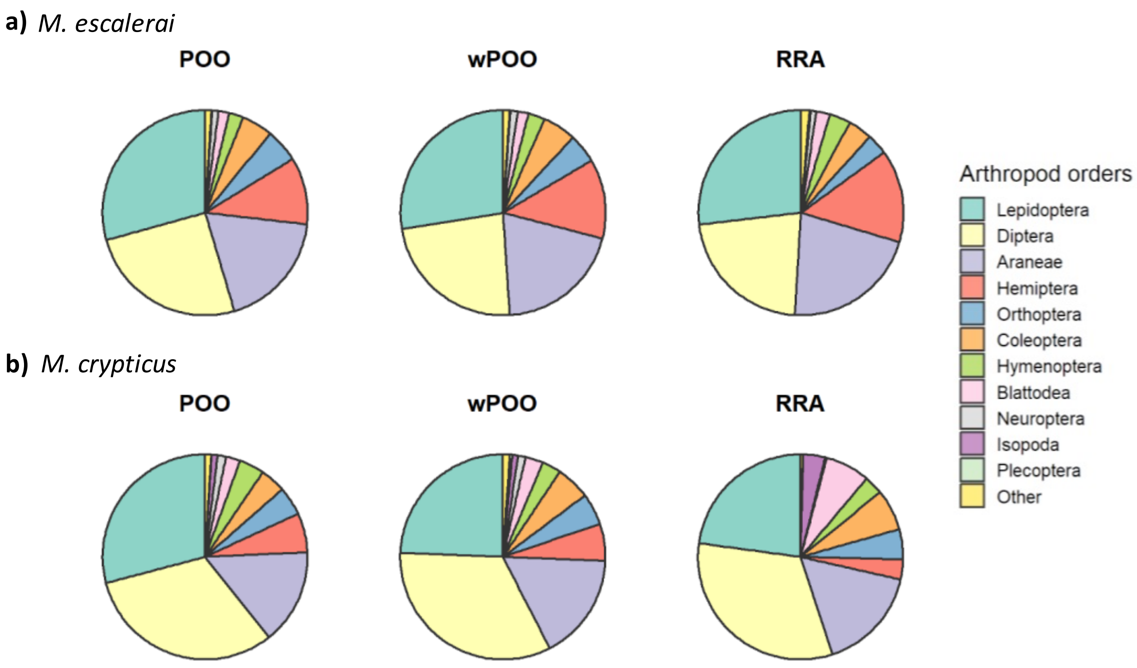

Fig. S2 – The diet of *Myotis escalerai* (a) and *Myotis crypticus* (b) according to percent frequency of occurrence (POO), weighted percent of occurrence (wPOO) and relative read abundance (RRA).

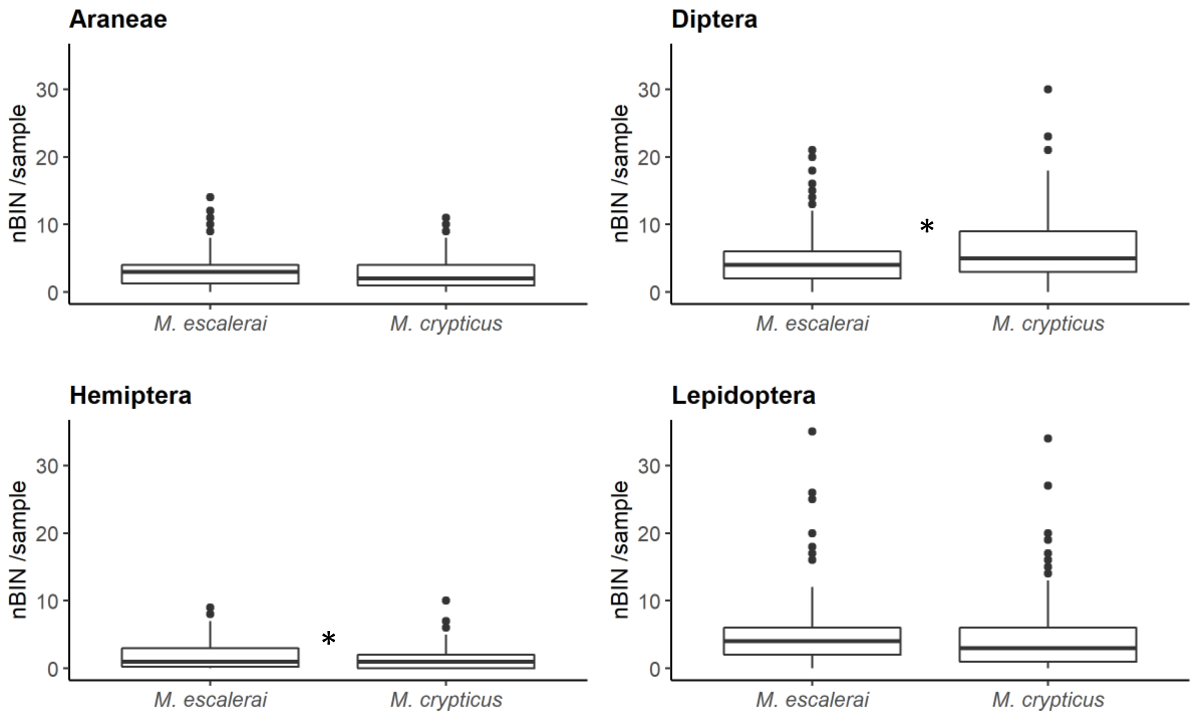

Fig. S3 – Number of BINs per sample of each of the four main Arthropod orders in the diet of *Myotis escalerai* and *Myotis crypticus*. Box represents first and third data quartiles. Whiskers extend from the hinge to the largest value no further than 1.5 times the distance between the first and third quartiles. Data beyond the end of the whiskers are plotted individually. Statistically significant differences are noted with a star.

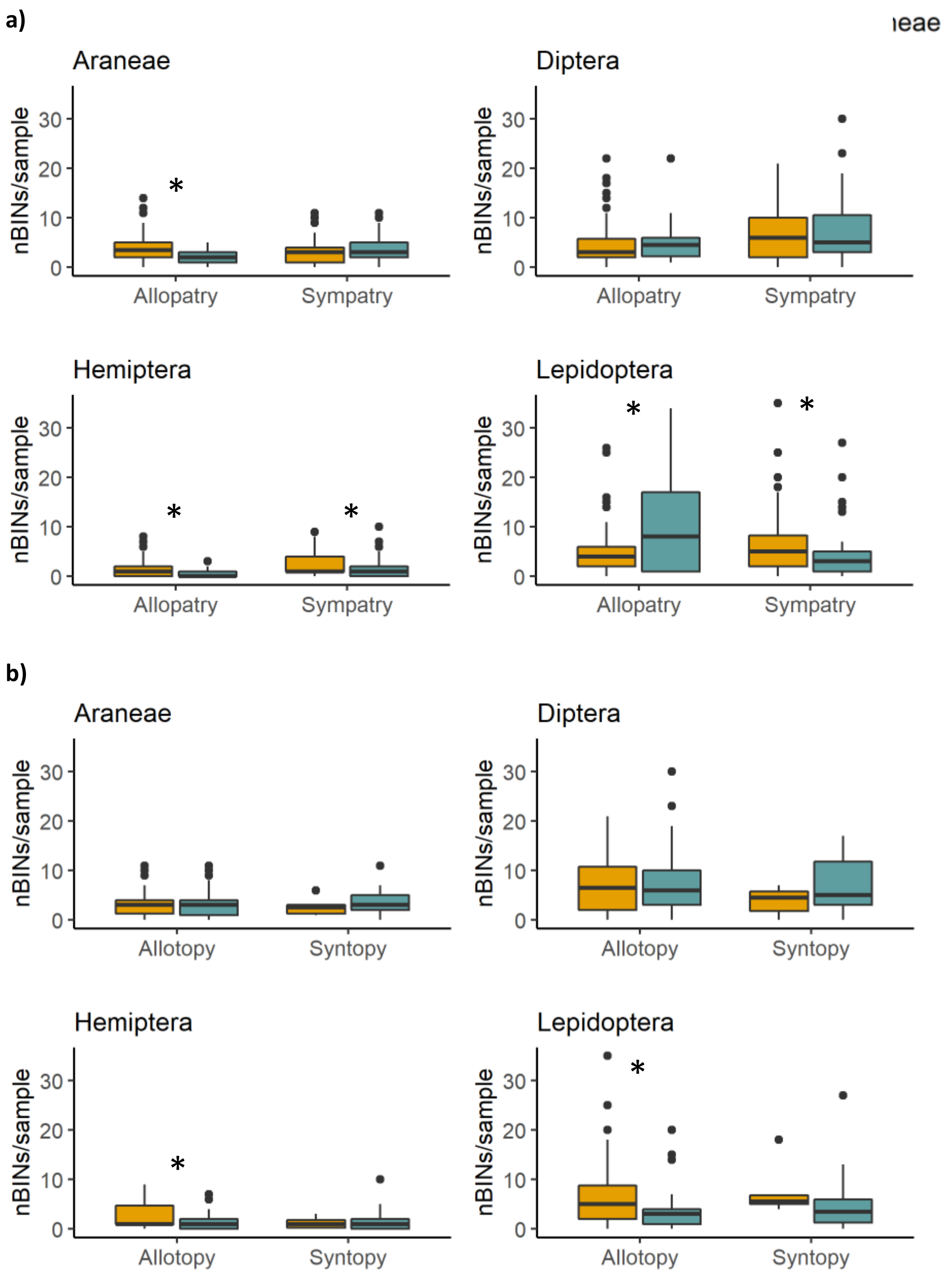

Fig. S4 – Number of BINs of each of the four main Arthropod orders in bat samples from sympatric and allopatric locations at the regional scale (a) and at fine scale (b). Statistically significant differences are noted with a star. Boxplot interpretation is detailed in Fig. S3.

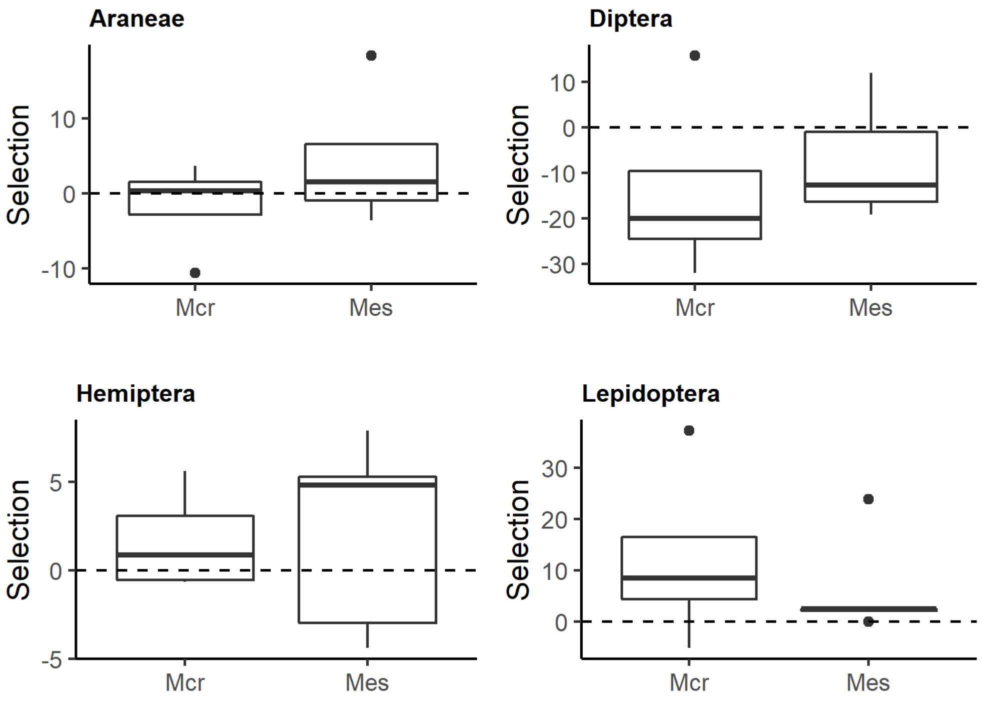

Fig. S5 – Selection values (bat diet wPOO – Arthropod availability) of *M. escalerai* and *M. crypticus* towards each of the main arthropod orders. Boxplot interpretation is detailed in Fig. S3.

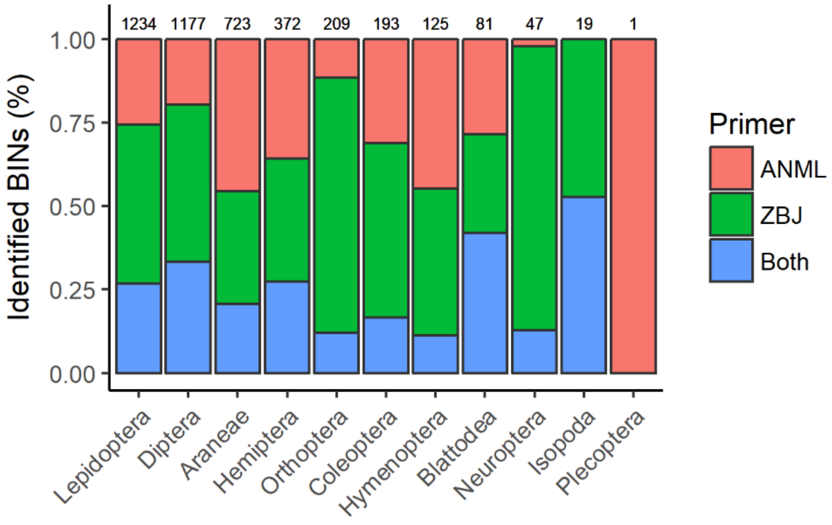

Fig. S6 – Number of BINs of each order. Portions coloured depending on the primer from which it was recovered. Red recovered uniquely by ANML, green uniquely by ZBJ, and blue recovered by both. Numbers above columns show number of BINs.

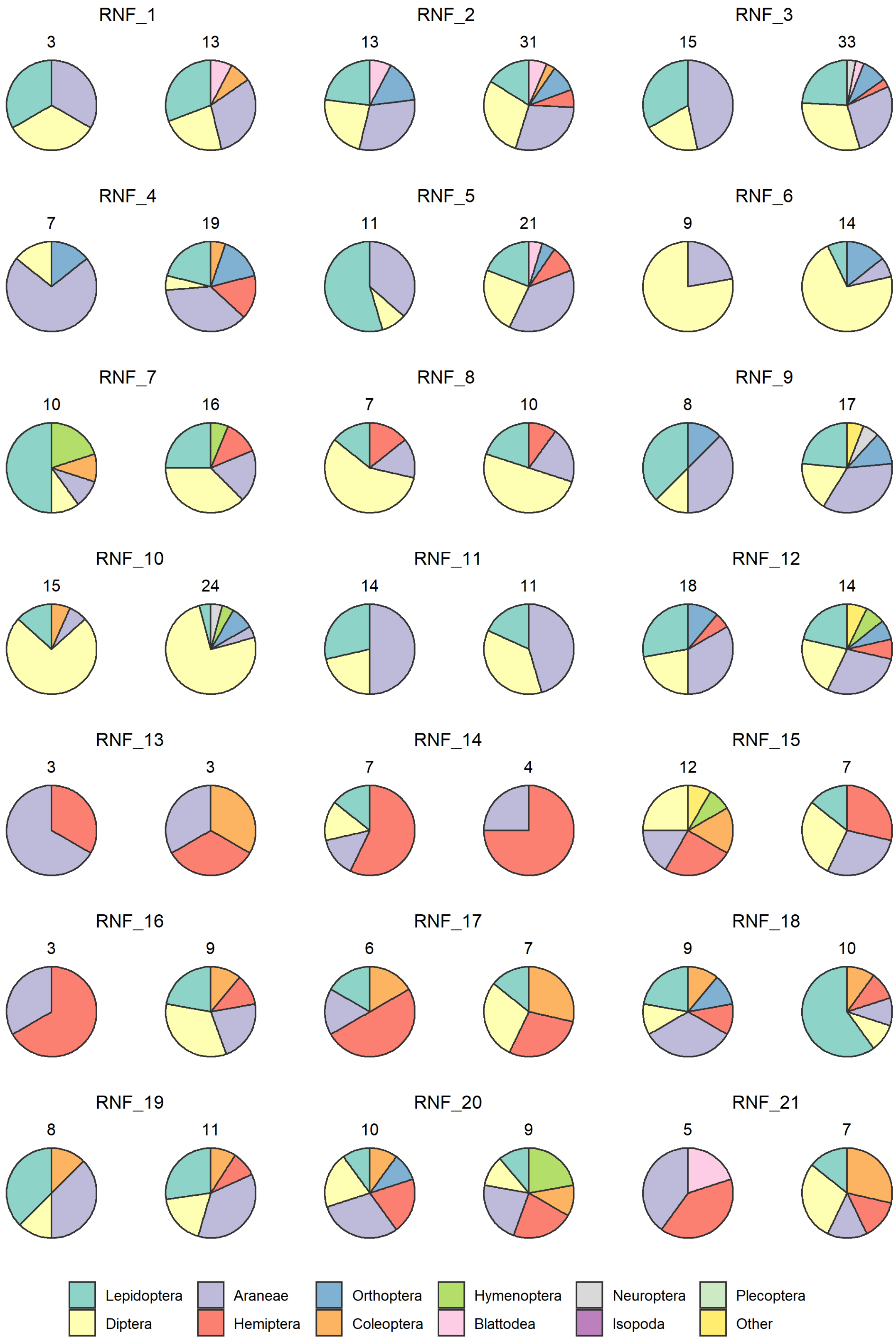

Fig. S7 – Prey order composition of a subset of individual dropping samples (RNF 1-21) comparing ANML (left) and ZBJ (right) primers. Number on top of each pie chart shows total BINs recovered.

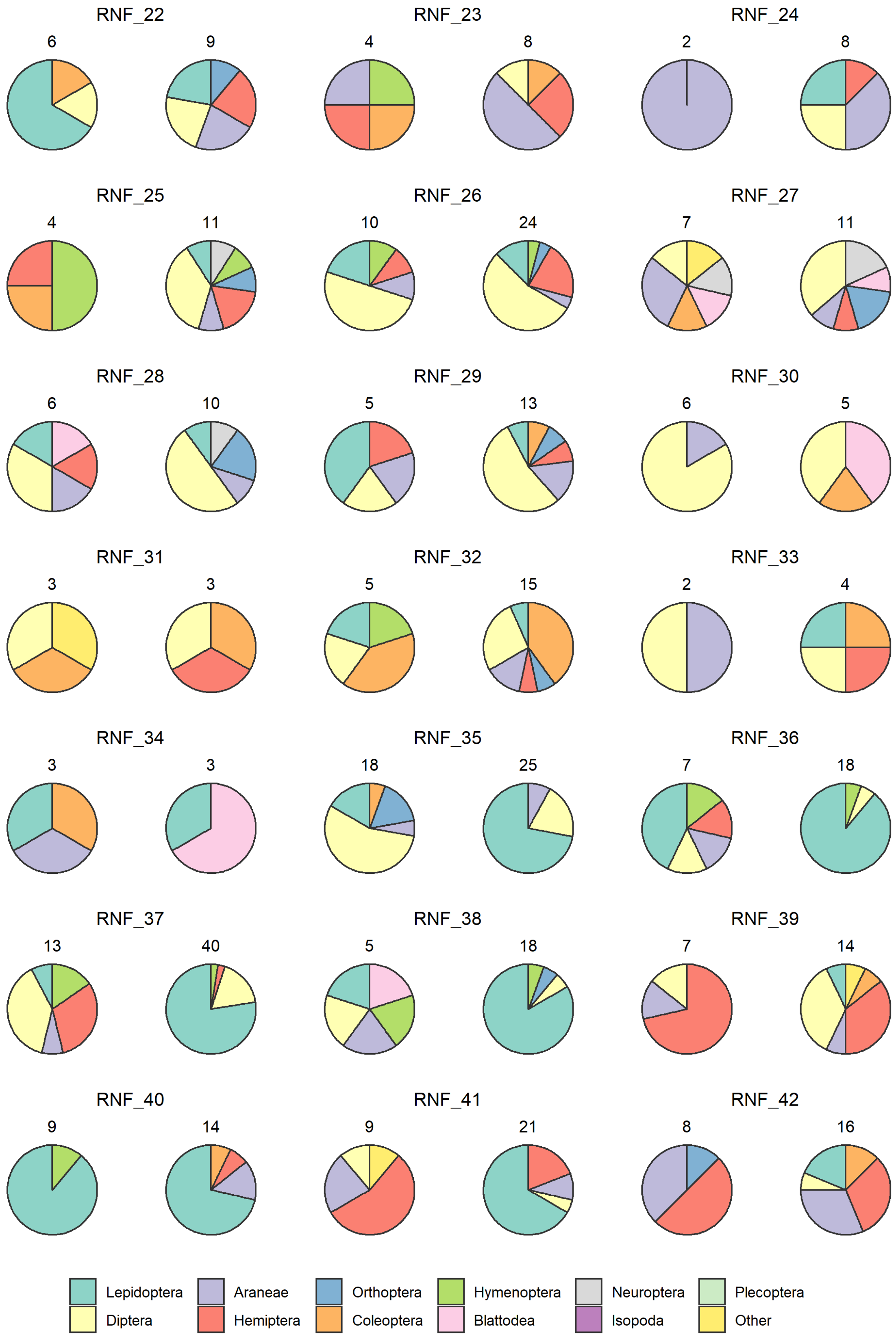

Fig. S8 – Prey order composition of a subset of individual dropping samples (RNF 22-42) comparing ANML (left) and ZBJ (right) primers. Number on top of each pie chart shows total BINs recovered.

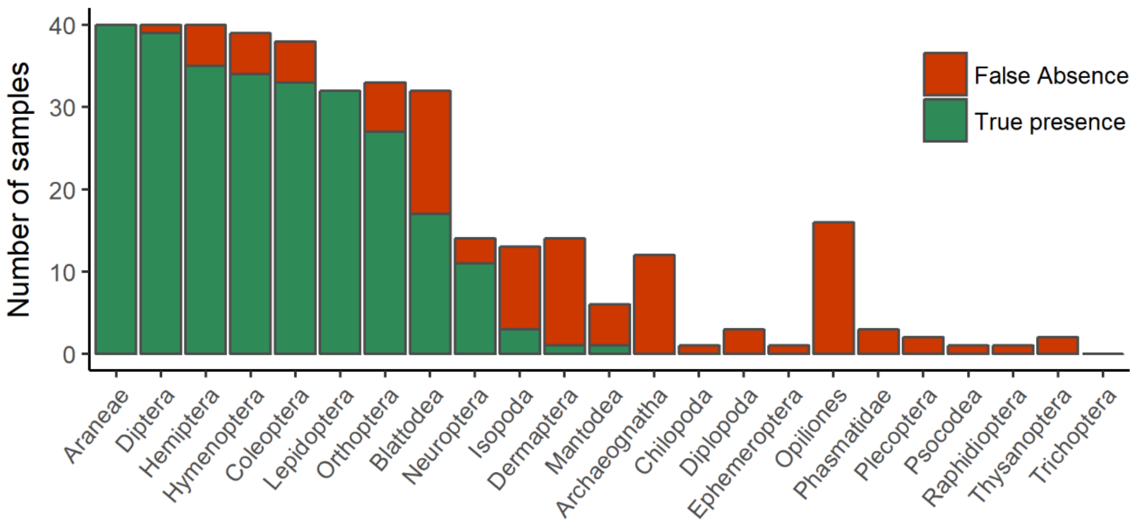

Fig. S9 – Number of samples in which each order appears in the morphological identification of sweeping samples (total bar height), and portion of those in which the order was present and absent in the subsequent molecular metabarcoding identification.

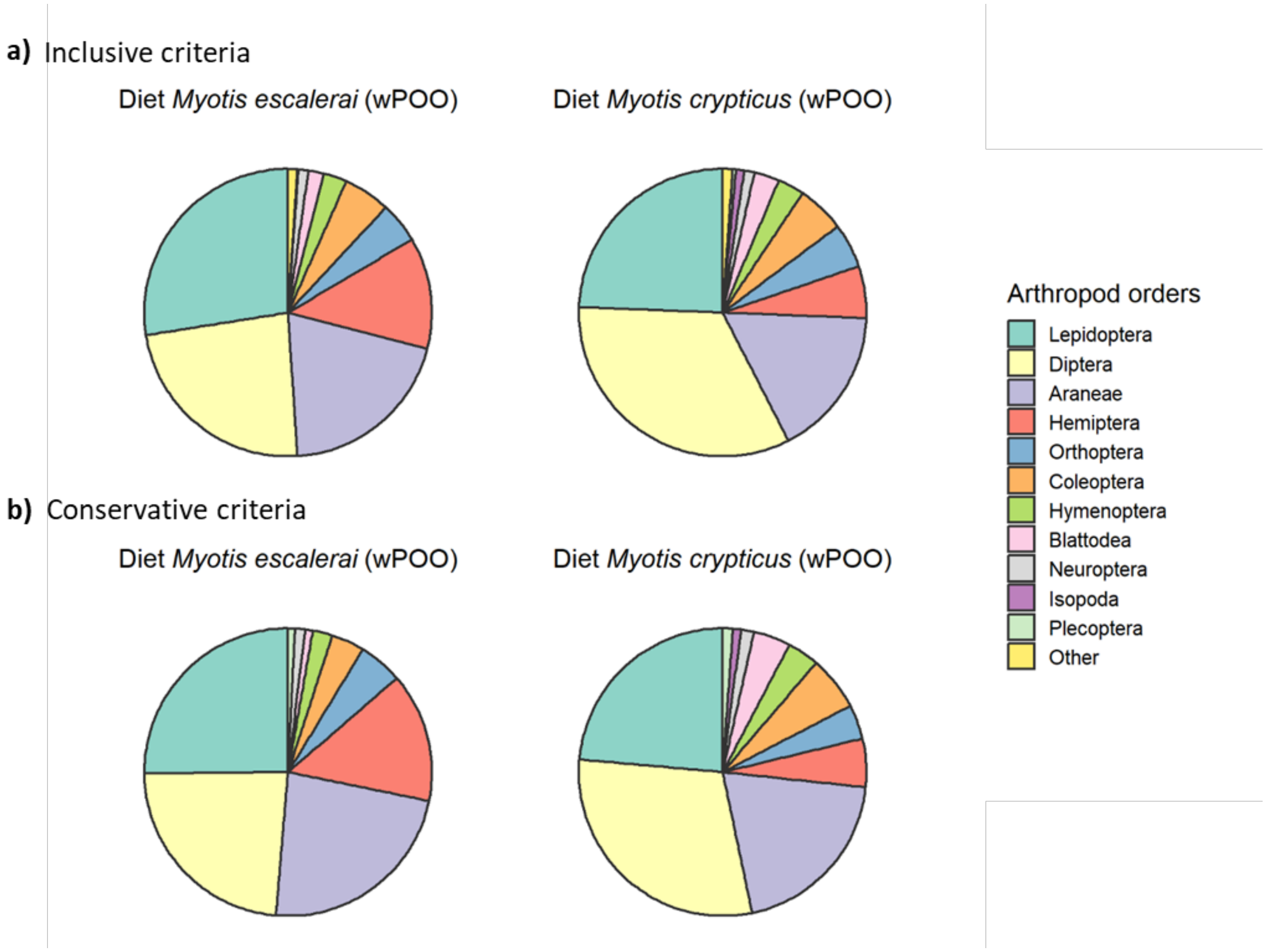

Fig. S9 – Overall diet composition of *M. escalerai* and *M. crypticus* using *w*eighted Percent of Occurrence (wPOO). Using the inclusive criteria (a) and the conservative union criteria (b) to pool prey composition from PCR replicates.
